# Mechanism-constrained D-optimal design of a flexible claw for peanut shelling under biological variability

**DOI:** 10.3389/fpls.2026.1831721

**Published:** 2026-08-17

**Authors:** Yuxuan Tuo, Yukun Pang, Xiwei Zhao, Mantuo Li, Yueshan Zhang, Zhiheng Yu, Tianwei Liang, Pengrui Zheng, Zhuoran Chen, Qiwen Sun, Xuebin Bai, Ying Zhao

**Affiliations:** School of Mechanical and Automotive Engineering, Liaocheng University, Liaocheng, China

**Keywords:** Hertzian-curved beam coupling, kernel damage, low signal-to-noise ratio, MC-DORSO, mean-normalized response, seed peanut, stress-concentration claw

## Abstract

**Introduction:**

Experimental optimization of seed-peanut shelling mechanisms is limited by the low signal-to-noise ratio of biological materials, because variations in pod size, shell thickness, shell-kernel clearance, and rupture behavior can mask the effects of claw geometry. This study proposes a flexible peanut shelling claw and a mechanism-constrained D-optimal response surface optimization (MC-DORSO) framework to reduce biological-variability-induced noise while lowering shelling force and kernel damage.

**Methods:**

A Hertzian-curved beam coupling model was used to guide the stress-concentration claw design. MC-DORSO combined mechanical feasibility filtering, D-optimal sampling, mean-normalized response modeling, and mixed discrete-continuous optimization. The relative shelling efficiency index *R_μ_* and adaptive stepwise regression were used to reduce baseline force differences among pod-size groups.

**Results:**

The optimized parameters were *θ* = 120°, *L_p_* = 16 mm, *γ* = 45°, and *H_t_* = 0.6 mm. In validation tests against flat-press compression, the optimized claw reduced mean critical shelling force by 38.82%, increased the intact-kernel rate from 84.00% to 94.00%, and increased the germination rate from 92.00% to 98.00%.

**Discussion:**

The proposed MC-DORSO-based claw design reduces the load required for single-pod shell rupture while improving kernel preservation, providing a scalable optimization approach for low-damage seed-peanut shelling.

## 1 Introduction

Peanut is an economically important oilseed crop, and its postharvest processing must preserve high kernel integrity, seed-coat integrity, and seed viability. Particularly, for seed peanuts, kernel cracking, seed-coat peeling, or embryo injury during shelling directly reduces germination rate and seed value. Previous studies have shown diverse modern peanut processing applications; however, peanut picking and shelling remain labor-intensive operations in the postharvest production chain Dey et al. (1999); Chen et al. (2023); Liao et al. (2024); Toomer (2018); Sun et al. (2017); Arya et al. (2016); Zhao et al. (2012). Therefore, reducing mechanical damage to kernels while maintaining shelling efficiency is a critical challenge for mechanized seed peanut shelling.

At present, peanut shelling equipment commonly uses a roller-concave screen structure, and the shelling process generally involves multiple mechanical actions, including impact, rubbing, extrusion, shearing, and tearing Liao et al. (2024). Such continuous shelling systems have high processing capacity and are suitable for batch processing of edible or oil-use peanuts. However, in continuous operation, collisions among peanut pods, repeated contact between peanut pods and the roller-concave screen, as well as local extrusion and friction often occur simultaneously. This multi-action process makes the stress state of individual pods difficult to control. For seed peanuts, this multi-contact, multi-load, and multi-particle coupling process can increase the risk of seed-coat damage, kernel cracking, and embryo injury, ultimately affecting seed germination performance Yu and Bao (2009); Blankenship and Person (1975); Wang et al. (2022); Xu et al. (2024).

Therefore, the design of low-damage seed peanut shelling mechanisms should not be based only on whole-machine high-throughput performance, but should also focus on the controlled shell rupture process of a single peanut pod. By studying the local rupture mechanism of a single pod under well-defined loading conditions, the effects of contact position, loading mode, geometric constraints, and individual size differences on the critical shelling force and kernel damage risk can be more clearly identified. A low-damage shelling unit developed on this basis can then be further extended to multi-unit array or continuous high-throughput shelling mechanisms.

For the optimization of peanut shelling mechanisms, previous studies have used finite element method (FEM), discrete element method (DEM), and physical parameter testing to analyze the force and rupture processes of peanut pods Wang et al. (2022); Xu et al. (2024); Mengchen et al. (2020). These methods provide useful references for understanding the overall stress state and optimizing shelling components. However, peanut pod shells are characterized by multilayer tissues, non-uniform fiber-bundle distributions, varying shell thickness and shell-kernel clearance, as well as substantial inter-pod variability. In the actual shelling process, crack initiation and propagation are affected not only by external loading, but also by local curvature, shell thickness, fiber structure, internal clearance, and loading position Aydin (2007); Akcali et al. (2006); Kurt and Arioglu (2018); Hartmond et al. (1996). Because existing simulation models usually require simplifications of material properties, geometric structures, and contact conditions, their results are more suitable for analyzing overall trends than for direct optimization of local crack initiation, shelling force, and kernel damage risk in low-damage shelling operations.

Response surface methodology (RSM) is commonly used for parameter optimization in agricultural machinery because it can establish empirical relationships between structural parameters and performance indicators through limited experiments Myers et al. (2016); Wang et al. (2022); Xu et al. (2024). Traditional response surface designs, such as Box-Behnken design and central composite design, usually generate experimental points within a regular factor space to fit a quadratic polynomial model Box and Behnken (1960); Myers et al. (2016). D-optimal design improves the information contribution of limited experimental points to model parameter estimation by maximizing the determinant of the information matrix, thereby reducing the number of experiments and improving parameter estimation efficiency Fedorov (1972); Atkinson et al. (2007); Meyer and Nachtsheim (1995). However, for a shelling claw with complex geometric coupling, the candidate space cannot be defined only by the independent upper and lower bounds of each design variable. Because the wedge angle, flat width, feeding opening size, protrusion height, protrusion mounting width, and seed-safety boundary constrain one another, some parameter combinations may satisfy the range of each individual variable but still be difficult to fabricate or assemble, unable to achieve stable feeding, or associated with potential kernel damage risk. Therefore, geometric feasibility screening must be performed before D-optimal sampling so that limited experimental points are concentrated in physically feasible and information-rich design regions.

In addition to geometric feasibility, experiments involving agricultural biological materials generally face large response dispersion and weak effective signals. Unlike homogeneous engineering materials such as metals and plastics, plant-based materials usually exhibit substantial biological variability, and their size, moisture content, maturity, tissue structure, local defects, and loading direction can all affect mechanical responses Mohsenin (2020); Sitkei (1986); Bourne (2002). This problem is especially pronounced in peanut shelling research. Peanut pod shells are natural biological composite structures developed from the ovary wall, with multilayer tissues, local fiber-bundle distributions, irregular curvature, and substantial individual size differences Bobet et al. (2020); Aydin (2007); Kurt and Arioglu (2018). Existing studies have also shown that pod size, shell thickness, shell-kernel clearance, moisture condition, and loading direction affect the compressive rupture behavior and critical shelling force of peanut pods Blankenship and Person (1975). If the absolute critical shelling force is directly used as the response variable for modeling, the true effect of claw geometric parameters may be masked by baseline force shifts caused by individual differences among peanut pods, reducing the ability of the response surface model to identify geometric effects.

Based on the above analysis, the optimization of a low-damage peanut shelling claw must address two issues simultaneously. First, the validity and information density of limited experimental points should be improved during the experimental-design stage so that the points are concentrated in physically feasible and modeling-relevant regions. Second, the baseline force shift caused by peanut size differences should be reduced during the data-analysis stage so that the response surface model can more clearly extract the influence trend of claw geometric parameters on relative shelling efficiency. To this end, this study proposes a flexible stress-concentration shelling claw for low-damage seed peanut shelling and establishes a mechanism-constrained D-optimal response surface optimization (MC-DORSO) framework. This framework integrates geometry-constrained D-optimal sampling, the mean-normalized relative shelling efficiency index *R_μ_*, response surface modeling, and mixed discrete-continuous parameter optimization to screen low-damage shelling claw parameter combinations within a representative range of size variation. Meanwhile, this study does not aim to directly evaluate the performance of a complete continuous shelling machine, but to establish a basic shelling unit and parameter optimization method that can be used for future array-type or continuous low-damage shelling mechanisms.

The main contributions of this study are as follows:

First, a Hertzian-curved beam coupling (HCBC) mechanistic model based on Hertz contact theory and curved-beam mechanics is proposed to describe the bending-dominated tensile rupture mechanism of peanut pod shells under local stress concentration, providing a mechanical basis for the geometric design of the stress-concentration shelling claw.
Second, a geometry-constrained D-optimal experimental design strategy is proposed. Feeding, orientation-adjustment, seed-protection, and protrusion-mounting constraints are embedded into the candidate-space filtering process, allowing D-optimal sampling to focus on physically feasible and information-rich design regions. This improves the information density of limited experimental points and provides a stronger basis for response surface modeling.
Third, a mean-normalized response modeling method based on *R_μ_* is proposed to reduce baseline force differences among peanut pods of different sizes and to extract the dominant influence trends of claw geometric parameters on relative shelling efficiency from low signal-to-noise ratio (SNR) biological-material experimental data.

## 2 Materials and methods

For clarity, the main geometric, mechanical, experimental-design, and response-modeling symbols used in this study are summarized in **Appendix A**.

**Figure 1:**
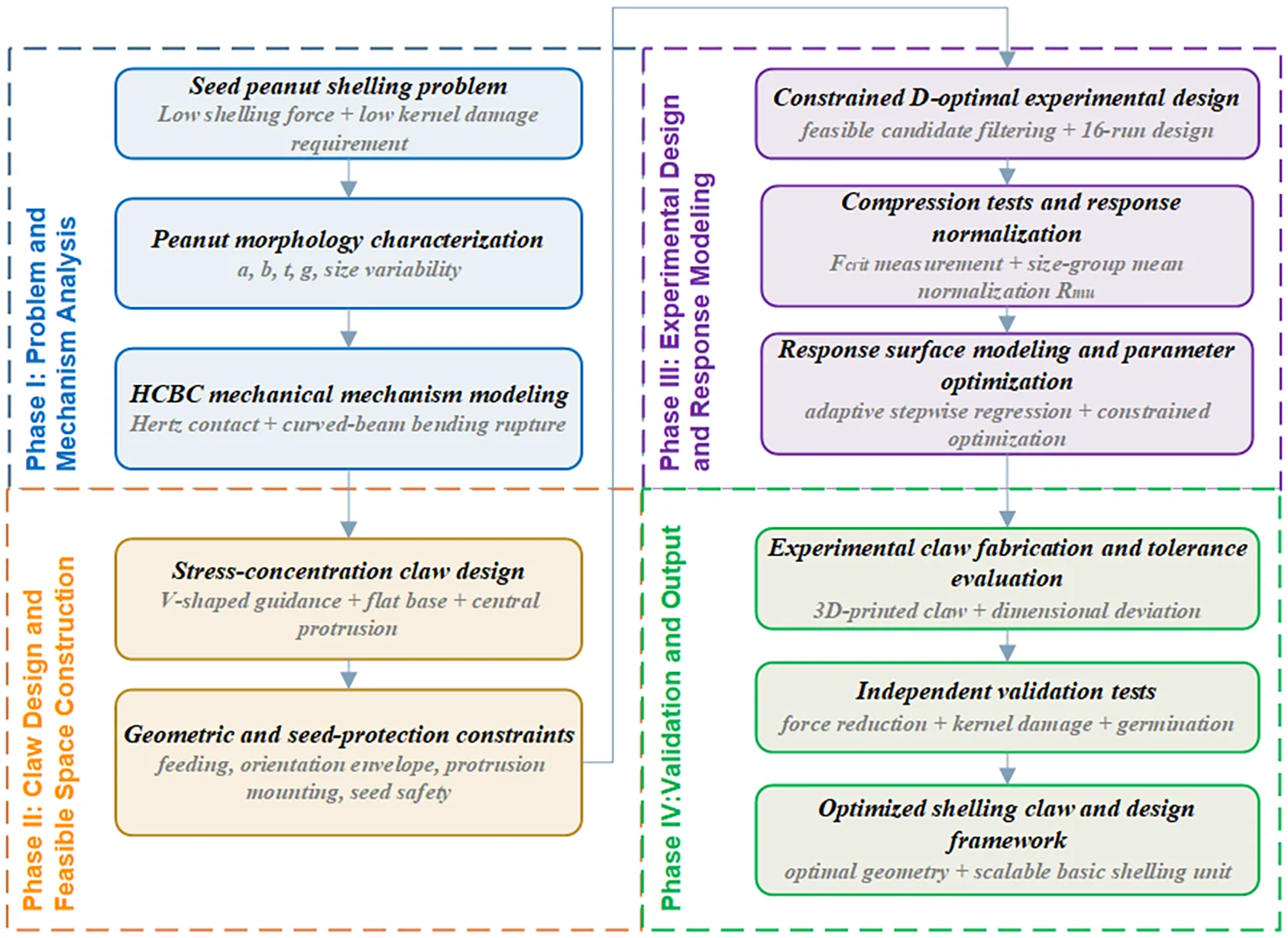
Overall workflow of the proposed MC-DORSO-based design and validation process for the flexible peanut shelling claw. The workflow links peanut morphology characterization, HCBC mechanism modeling, stress-concentration claw design, feasible-space construction, constrained D-optimal experimental design, response normalization and modeling, parameter optimization, and independent validation tests.

The overall workflow of this study is shown in **Figure 1**. The study was organized into four stages: problem and mechanism analysis, claw design and feasible-space construction, experimental design and response modeling, and validation and output.

### 2.1 Mechanical mechanism model for the compression-induced rupture of peanut shells

**Figure 2:**
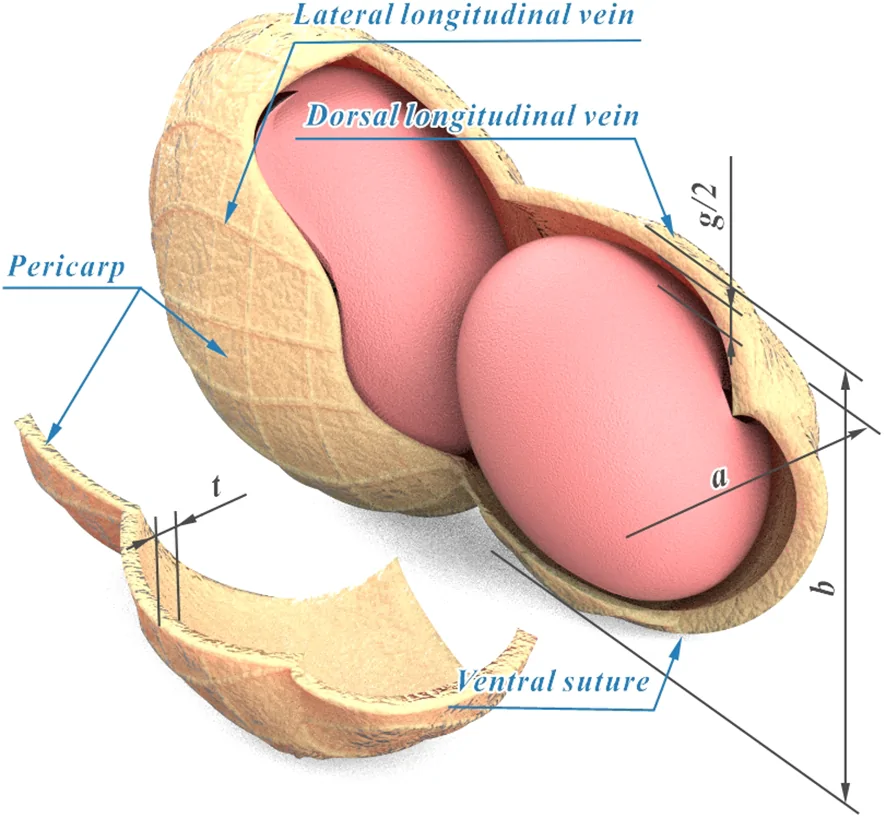
Structural composition and geometric definitions of the peanut pod. The diagram illustrates the pericarp structure, longitudinal veins, and ventral suture. Key geometric parameters include the minor axis (*a*), major axis (*b*), shell thickness (*t*), and shell-kernel half-gap (*g*/2), which form the basis for the subsequent mechanical modeling.

The peanut pod shell is a multilayer biological structure developed from the ovary wall, mainly consisting of outer, middle, and inner tissue layers. Its surface contains longitudinal and transverse veins, and the ventral suture forms a structurally weak region related to crack initiation. Because shell thickness, local curvature, vascular-bundle distribution, and shell-kernel clearance affect the mechanical response of the pod, these features were considered in the subsequent mechanical modeling. The main structural features and geometric parameters are shown in **Figure 2**.

Conventional flat-press shelling mainly applies normal pressure to the peanut pod and induces whole-pod compression before effective shell rupture. This loading mode was used here as a mechanical baseline because it represents shell rupture without a designed local stress-concentration structure. Previous work showed that normal loading near the ventral suture can produce stress concentration and fracture near the apical region of the pod Liu et al. (2012).

**Figure 3:**
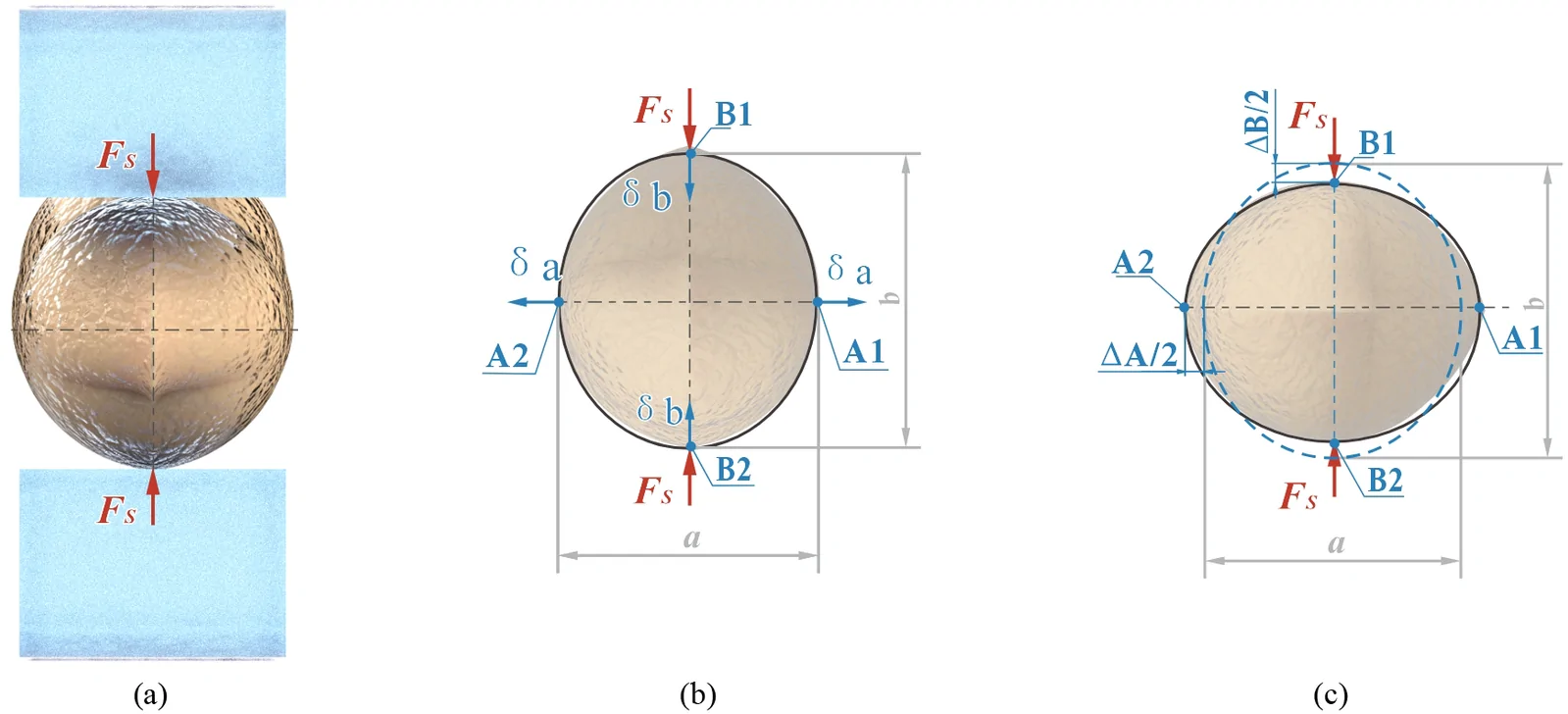
Compression-induced deformation mechanism of a peanut pod under conventional flat-press loading: **(a)** quasi-static flat-press loading configuration; **(b)** simplified elliptical cross-sectional model showing the force-induced deformation directions; **(c)** deformed cross-section showing lateral expansion Δ*A*/2 and vertical shortening Δ*B*/2 relative to the initial contour.

*Note.* **Figure 3a** shows the quasi-static flat-press compression configuration, where the peanut pod is compressed by two opposite forces *F_s_* along the *B*_1_–*B*_2_ direction. **Figure 3b** shows the simplified elliptical cross-sectional model before deformation; *a* and *b* denote the minor- and major-axis dimensions, respectively, and *A*_1_/*A*_2_ and *B*_1_/*B*_2_ represent the corresponding axis endpoints. **Figure 3c** shows the deformed cross-section after compression. The dashed contour represents the initial pod profile, whereas the solid contour represents the compressed profile; Δ*A*/2 and Δ*B*/2 indicate the lateral expansion and vertical shortening on each side, respectively.

As illustrated in **Figure 3**, when a normal force *F_s_* is applied parallel to the ventral suture plane *s*, where *s* denotes the plane passing through the ventral suture and the longitudinal axis of the pod, the endpoints of the major axis *b* undergo an inward deformation *δ_b_*, while an outward deformation *δ_a_* occurs along the minor axis *a*. This deformation pattern provides the reference case for comparing the proposed stress-concentration claw with conventional whole-pod compression.

**Figure 4:**
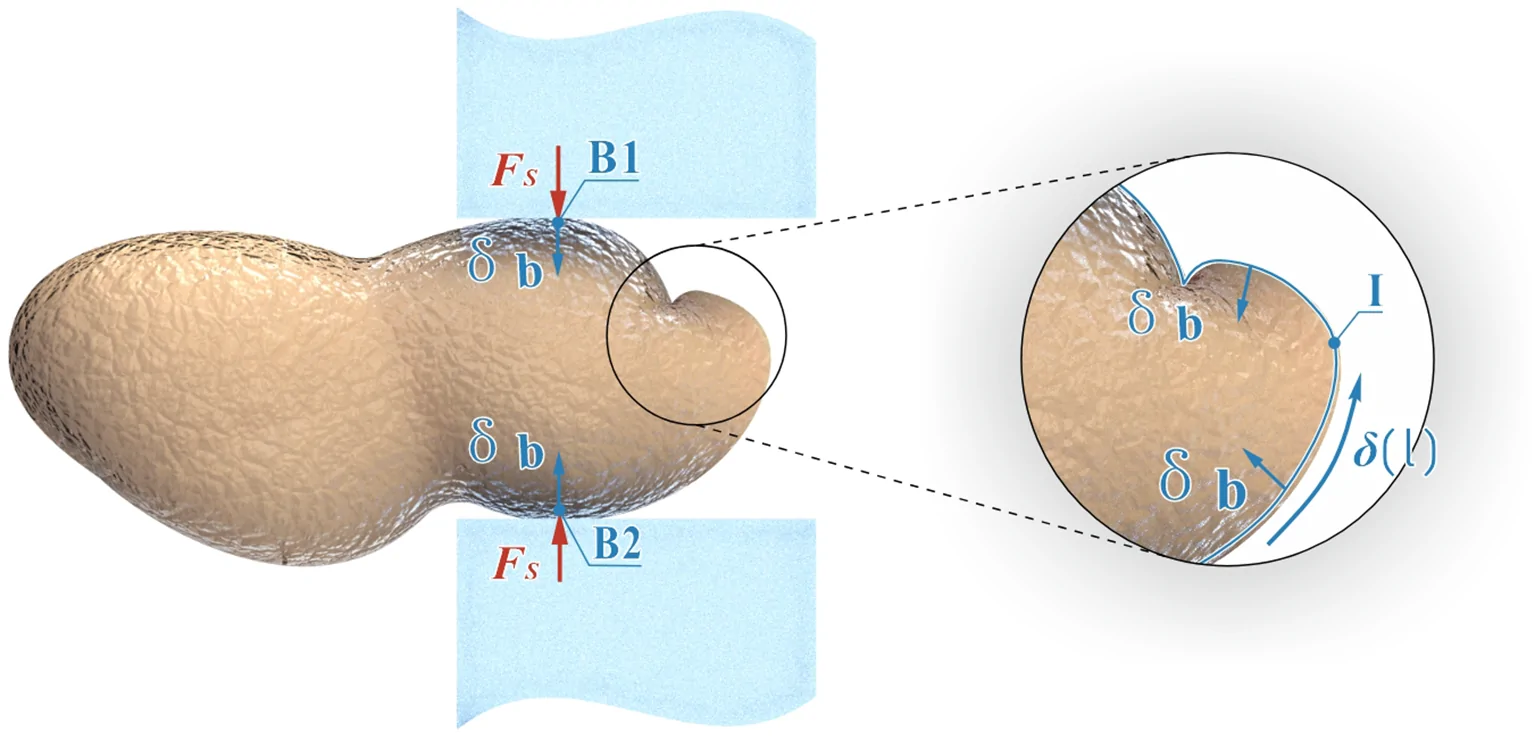
Peanut shelling mechanism diagram.

As illustrated in **Figure 4**, from a lateral perspective, the pod’s smooth cross-sectional profile causes the stress concentrated at the endpoints of the *b*-axis to diminish gradually as it propagates along the contour. Concurrently, an induced deformation *δ_a_* extends toward the pod’s apex *l*. A similar deformation *δ_b_* propagates along the *a*-axis, also converging at apex *l*.

**Figure 5:**
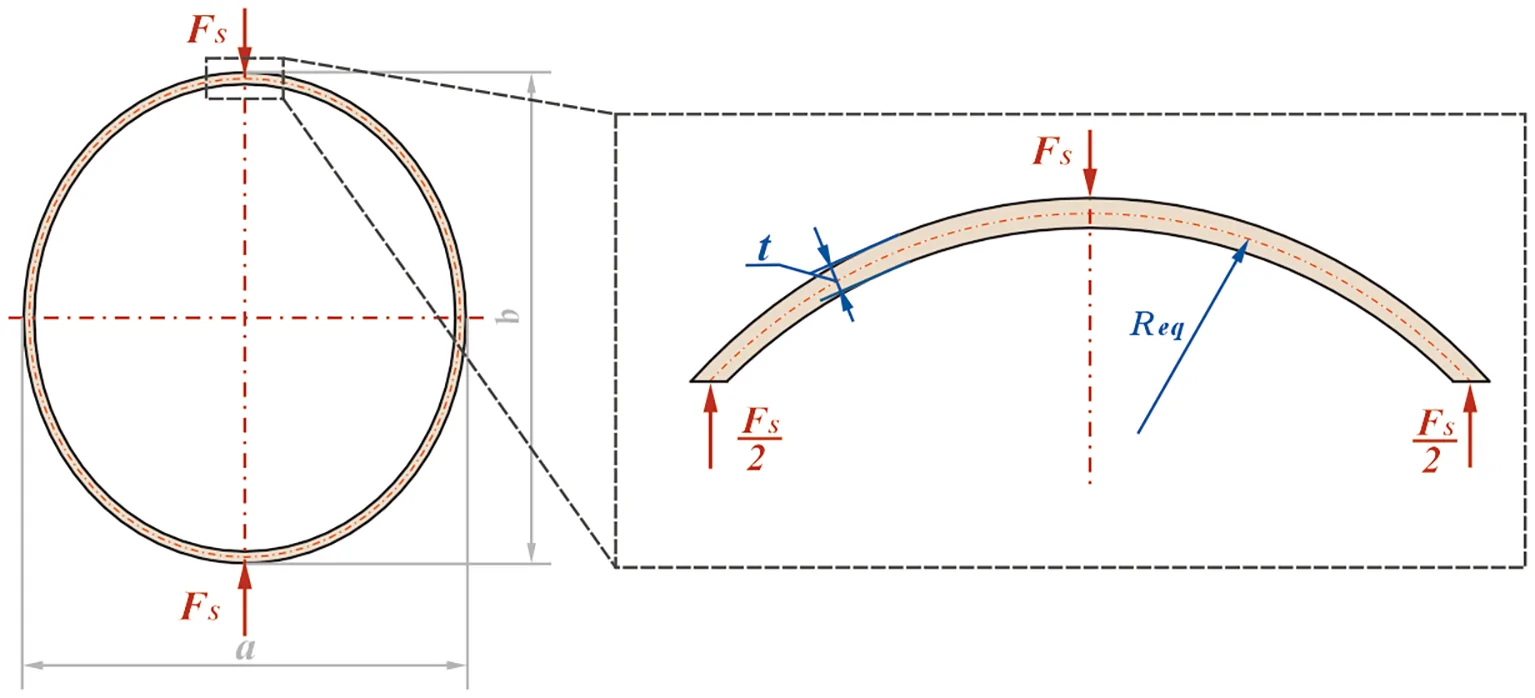
Schematic of the local thin-walled curved-beam idealization near the loading apex.

As shown in **Figure 5**, the local region of the peanut shell near the loading apex can be idealized as a thin-walled curved-beam structure subjected to the radial shelling force *F_s_*. Here, *F_s_* denotes the total externally applied shelling force, whereas the local curved-beam stress is evaluated per unit shell width by introducing the equivalent radial load per unit width, *q_s_* = *F_s_*/*w_s_*, where *w_s_* is the effective shell width involved in local deformation.

The bending behavior of this local shell segment is characterized by the equivalent local curvature radius *R*_eq_ near the loading region. Under approximately symmetric loading conditions, the equivalent radial load per unit width *q_s_* is mainly transmitted through the shell segments on both sides of the loading region, with each side carrying approximately *q_s_*/2 of the compressive force flow.

Therefore, calculated per unit shell width, the direct compressive stress near the inner wall can be approximated as (Equation 1)

(1) $$\sigma_{N}\approx \frac{q_{s}/2}{t}=\frac{q_{s}}{2t}.$$

Here, σ*_N_* represents the equivalent direct compressive stress near the inner wall of the local shell segment, *q_s_* is the equivalent radial load per unit shell width, and *t* is the average shell thickness. Because the load is assumed to be transmitted approximately symmetrically through the shell segments on both sides of the loading region, each side carries approximately *q_s_*/2 of the compressive load.

Meanwhile, the local curved shell undergoes bending deformation under the radial load, inducing circumferential tensile stress on the inner wall. For a thin-walled section with unit shell width, this bending stress can be expressed using the beam-bending relation as (Equation 2)

(2) $$\sigma_{M}=\frac{6m_{s}}{t^{2}}$$

where *m_s_* denotes the bending moment per unit shell width.

To further specify the bending term, this study refers to the analytical concepts of elastic elliptical rings and non-circular curved beams Mitchell (1968). The small shell segment near the loading apex of the peanut pod is idealized as a thin-walled curved beam with an equivalent local curvature radius *R*_Eq._ The bending stress in this local shell segment is mainly governed by the equivalent radial load per unit width *q_s_*, the equivalent local curvature radius *R*_eq_, the shell thickness *t*, and the local boundary conditions. Therefore, it can be written as (Equation 3)

(3) $$\sigma_{M}\approx \zeta \frac{q_{s}R_{eq}}{t^{2}}$$

Here, *ζ* is a dimensionless local bending geometry coefficient that characterizes the influence of local curvature, contact boundary conditions, and load-transfer mode on the magnitude of the bending stress.

Accordingly, the circumferential stress on the inner wall of the loading region can be expressed as the superposition of direct compressive stress and bending-induced tensile stress (Equation 4):

(4) $$\sigma_{\text{in}}\approx -\sigma_{N}+\sigma_{M}=-\frac{q_{s}}{2t}+\zeta \frac{q_{s}R_{eq}}{t^{2}}$$

In this expression, the first term, $-q_{s}/\left(2t\right)$, represents the direct compressive stress induced by the equivalent radial load per unit width in the local shell segment, with the negative sign denoting compression. The second term, $\zeta q_{s}R_{\text{eq}}/t^{2}$, represents the circumferential tensile stress induced on the inner wall by the bending of the local curved shell. By introducing the equivalent local curvature radius *R*_eq_ near the loading region, this expression characterizes the bending-induced tensile stress effect of the local elliptical shell section of the peanut pod under compression.

Because the shell is a typical thin-walled structure (*t* ≪ *R*_eq_), the bending term scales inversely with the square of the thickness (1/*t*^2^), whereas the direct compression term scales with 1/*t*. This indicates that the bending-induced tensile component can dominate the local stress state near the loading region.

**Figure 6:**
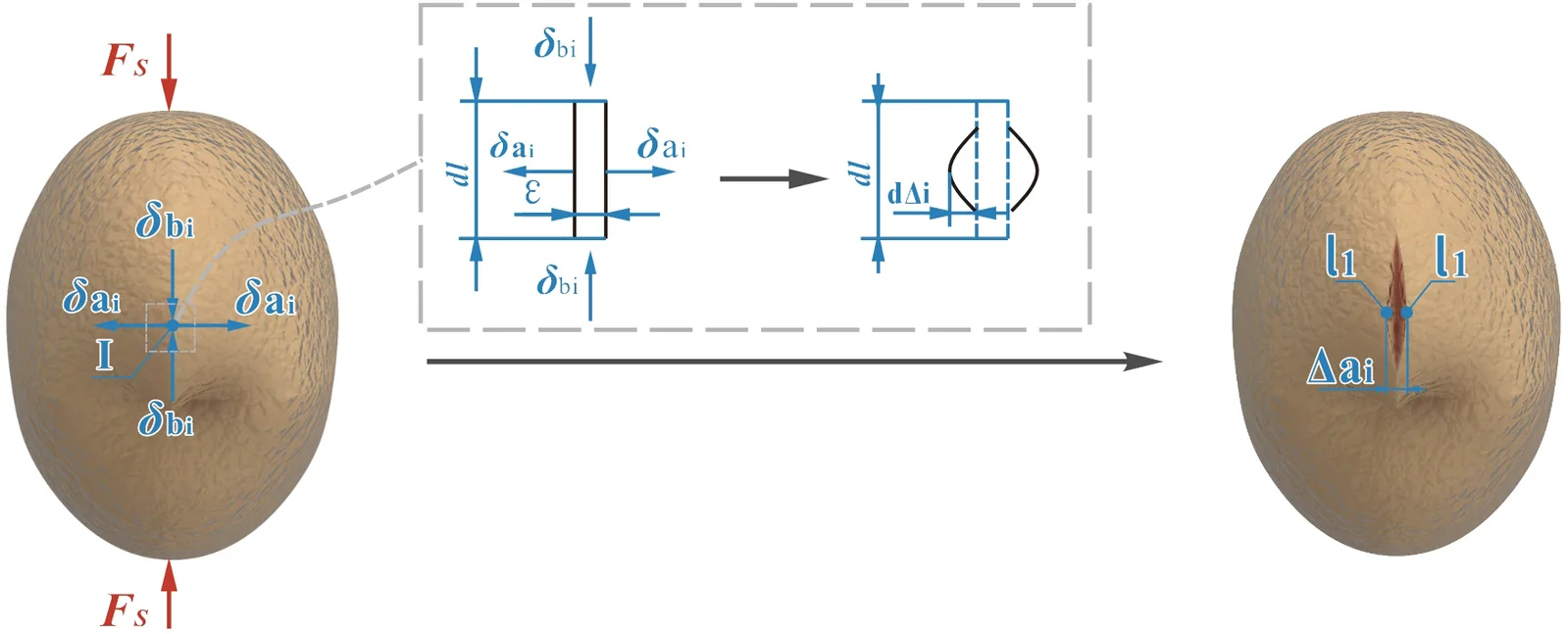
Peanut shelling mechanism: fracture of the apical differential element.

As shown in **Figure 6**, under external loading, this deformation-induced bending tension may overcome the initial compressive effect. Once the resultant inner-wall tensile stress exceeds the interlaminar bond strength σ_bond_ of the fibrous layer, the differential element undergoes Mode I opening fracture.

Macroscopically, this causes fracture initiation at apex *l*, followed by crack propagation along the ventral suture, ultimately achieving shell rupture.

To establish a computable mechanical model for geometric design and parameter optimization, the peanut pod was simplified as an elliptical thin-shell structure with representative major and minor axes, average shell thickness, and an equivalent local curvature radius.

This simplification does not aim to predict the complete three-dimensional crack path of each individual pod. Instead, it extracts the dominant relationships among claw geometry, local contact curvature, stress concentration, and bending-dominated shell rupture. Similar reduced-order shell approximations are commonly used to describe the dominant bending behavior of complex curved structures while acknowledging the influence of boundary conditions and material heterogeneity Zhai et al. (2026).

Given the pod’s approximately elliptical cross-section, differential geometry indicates that the local mechanical response at the loading point (typically the ventral suture) depends on its geometric curvature. Consequently, an equivalent radius of curvature, *R*_eq_, is introduced to refine the geometric model (Equation 5):

(5) $$R_{\text{eq}}=\frac{\rho_{major}^{2}}{\rho_{minor}}\approx \frac{a^{2}}{2b}$$

where *a* and *b* denote the minor and major axes of the pod cross-section, respectively. The above equation holds when the external load is applied at the major axis endpoints. Subsequently, *R*_eq_ is utilized to characterize the shell’s local geometric stiffness.

Initially, the contact behavior between the loading object and the shell is evaluated. Based on Hertz contact theory, under an applied normal load *F*, the maximum pressure *p*_max_ generated within the contact zone is given by (Equation 6):

(6) $$p_{\max\;\;}=\sqrt{\frac{FE^{*}}{\pi R^{*}L}}$$

where *F* is the applied normal force, E^*^ is the equivalent elastic modulus of the contact pair, and R^*^ is the relative radius of curvature. R^*^ satisfies $1/R^{*}=1/R_{\text{ind}}+1/R_{\text{eq}}$, where *R*_ind_ denotes the curvature radius of the loading object’s contact surface.

Consequently, the loading object’s geometry (*R*_ind_) affects the contact stress distribution. As the loading surface transitions from a flat plane ( $R_{\text{ind}}\to \infty$) to a sharp protrusion ( $R_{\text{ind}}\to 0$), the local maximum pressure *p*_max_ increases substantially. This mechanism provides a theoretical basis for minimizing the energy required for shelling.

### 2.2 Design of the shelling claw based on a controllable stress concentration mechanism

Consequently, achieving low-damage shelling requires a loading strategy that minimizes axial compression (the first term in Equation 4, which risks kernel damage) while maximizing the local bending moment (the second term in Equation 4, which promotes fracture). Specifically, reducing the loading object’s curvature radius *R*_ind_ amplifies this local bending effect. This principle forms the theoretical basis for the stress-concentration claw developed in this study.

**Figure 7:**
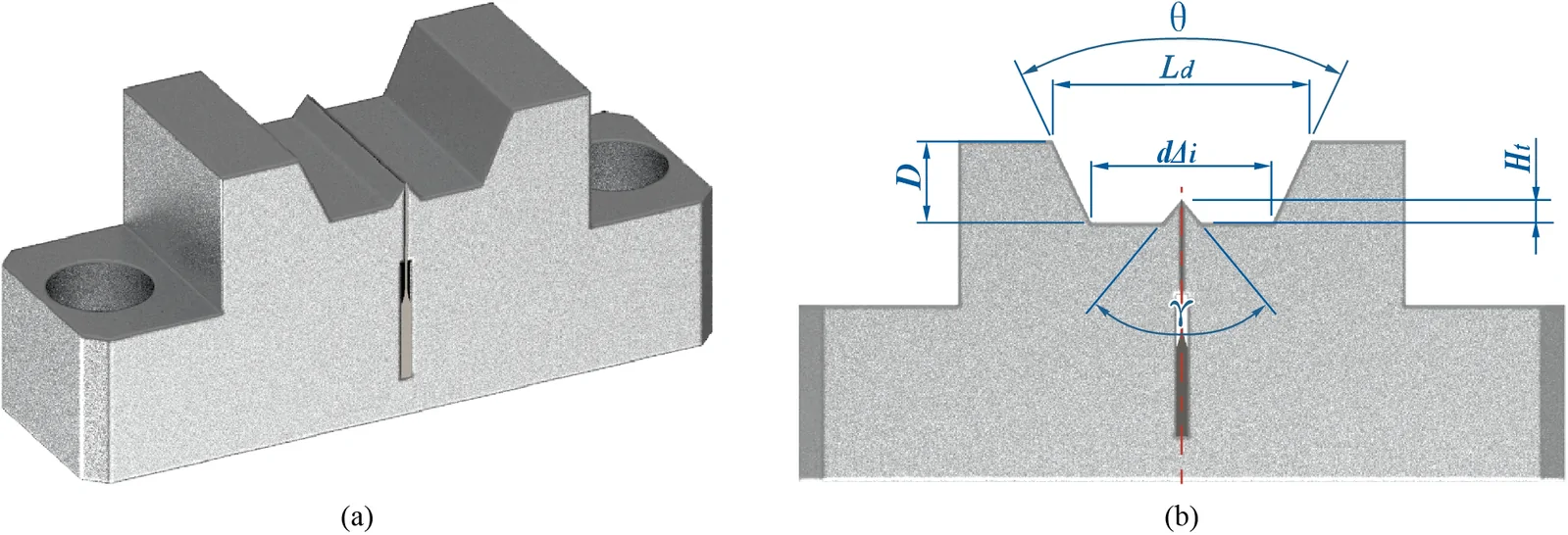
Structure and geometric parameter definitions of the gripping claw: **(a)** isometric view of the gripper; **(b)** dimensional drawing of the gripper.

As pressure is applied, the stress concentrates and narrows toward point *I*. Based on this mechanism, a novel shelling claw is designed to induce controllable stress concentration directly along the ventral suture. This pre-induced concentration accelerates fracture initiation at the apical region, thereby minimizing the required compression stroke and protecting the internal seeds. For this mechanism to be effective, the structural protrusions must precisely target the ventral suture and apply a normal force parallel to its plane, driving rapid crack propagation. The finalized claw geometry is illustrated in **Figure 7**.

As illustrated in **Figure 7a**, the claw’s distal ends feature bilateral V-shaped openings to stabilize the peanut during transport and compression, ensuring the ventral suture plane aligns precisely with the tool’s centerline. The bottom incorporates a flat surface with a central triangular protrusion. A slotted blade with an exposed tip is mounted at the protrusion’s apex to apply controllable stress concentration directly along the ventral suture. The corresponding geometric parameters for this structure are defined in **Figure 7b**.

#### 2.2.1 Modeling of probabilistic constraints based on biological variability

**Figure 8:**
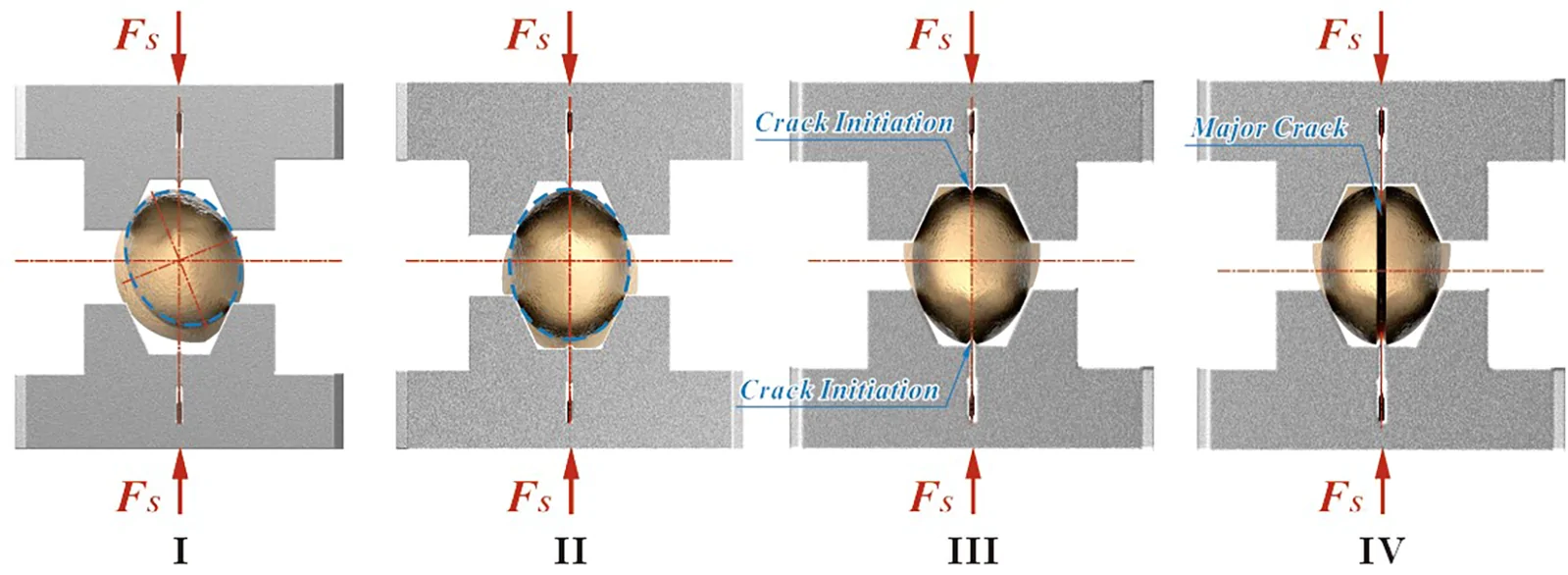
Schematic diagram of the staged compression process of the gripping claw.

Prior to establishing the parametric mechanical model, the compression sequence of the peanut pod in the gripping claw was analyzed. As illustrated in **Figure 8**, this process comprises four consecutive stages:

Stage 1: V-groove guidance and orientation. The peanut contacts the bilateral V-shaped inclined surfaces, which guide and complete its spatial alignment.
Stage 2: Lateral constraint and stiffening. As the total gripping force *F_s_* increases, the V-groove envelops the pod to a depth *D*. The lateral compression force *F_x_* rises rapidly, imposing strong constraints on the shell and consequently increasing its equivalent constraint stiffness *K*_shell_.
Stage 3: Protrusion contact and stress concentration. Following stabilization, continued loading presses the sharp protrusion into the shell. The force *F_s_* redistributes between the flat surface *L_p_* and the protrusion height *H_t_*, causing the local stress σ_max_ to concentrate rapidly at the tip.
Stage 4: Rupture guidance. Once the local tensile stress at the stress-concentration tip exceeds the interlaminar bond strength σ_bond_, an initial crack is triggered. The apex angle *γ* then exerts a wedge-splitting effect, driving crack propagation to achieve complete shell rupture.

**Figure 9:**
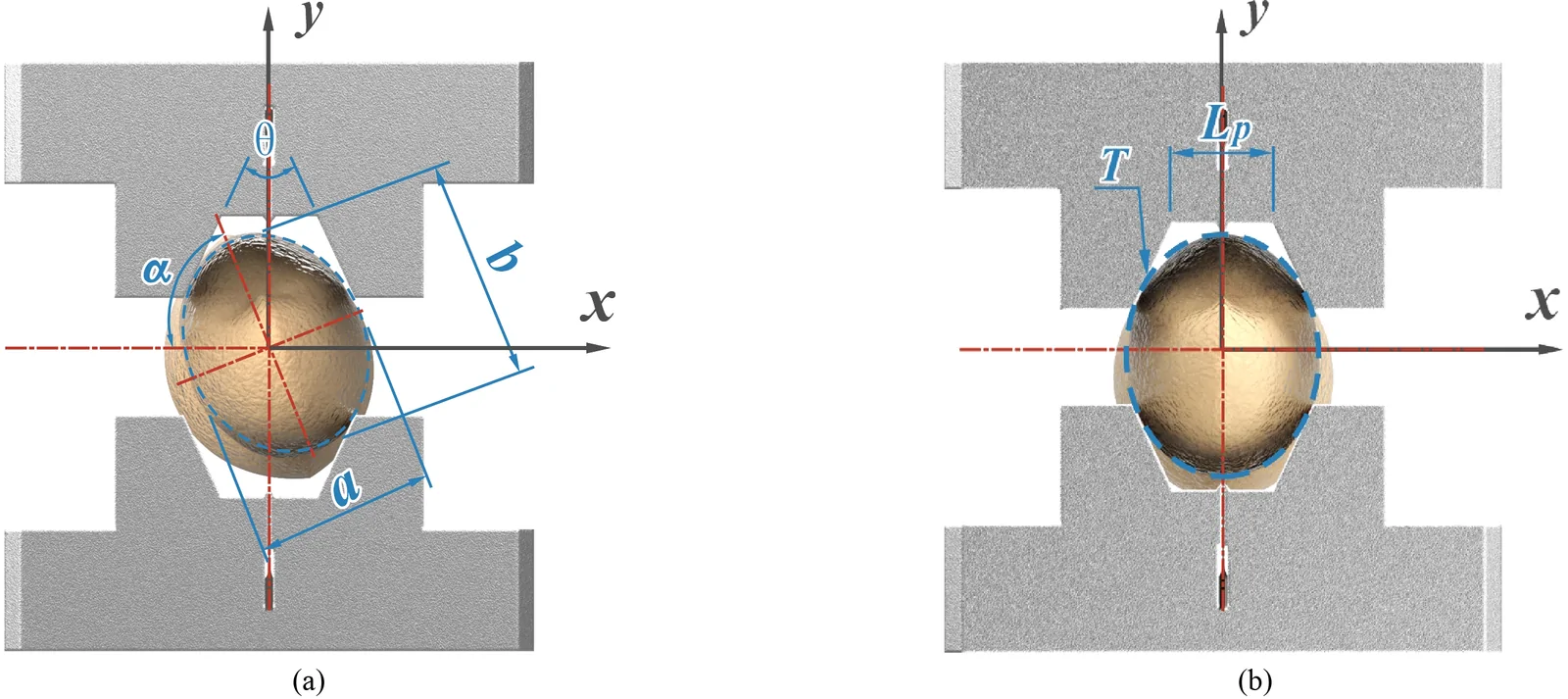
Schematic diagram of peanut orientation adjustment by inclined surfaces: **(a)** Pre-adjustment state; **(b)** Post-adjustment state.

During Stages 1 and 2, the primary mechanical functions of the V-shaped inclined surfaces are orientation guidance and lateral constraint. As illustrated in **Figure 9**, during actual compression, the bilateral inclined surfaces restrict the pod’s outward deformation along the *a*-axis. This structural interference constitutes the “rigid constraint” effect, wherein the increase in *K*_shell_ is synergistically governed by the wedge angle *θ* and the flat surface width *L_p_*. Consequently, the mechanical coupling between these two parameters warrants detailed analysis.

Assuming a fixed *L_p_*, the applied gripping force *F_s_* resolves into a normal force *F_N_* and a lateral compression force *F_x_* along the inclined surfaces. By neglecting friction and establishing a quasi-static force balance model, we obtain (Equations 7, 8):

(7) $$F_{N}=\frac{F_{s}}{2\sin(\theta /2)}$$

(8) $$F_{x}=F_{N}\cos\;(\theta /2)=\frac{F_{s}\cos(\theta /2)}{2\sin(\theta /2)}=\frac{F_{s}}{2\text{tan }(\theta /2)}$$

According to this mathematical model, as the wedge angle *θ* decreases, tan(*θ*/2) approaches zero, causing the lateral compression force *F_x_* to tend toward infinity. This extreme condition, characterized by a force surge due to an undersized *θ*, is defined herein as “strong restriction”.

Furthermore, assuming a constant *θ*, the flat surface width *L_p_* also significantly influences *F_x_*. During actual compression, the pod’s *b*-axis endpoints must be pressed to the claw’s base. Because *L_p_* dictates the separation between the bilateral inclined surfaces, a narrower *L_p_* necessitates a greater gripping force *F_s_* to fully seat the peanut. This elevated *F_s_* proportionally amplifies *F_x_*, thereby intensifying the rigid constraint effect.

Concurrently, the V-shaped inclined surfaces facilitate spatial alignment. Should the pod experience a rotational offset around its centroid, these surfaces correct its posture, ensuring the ventral suture aligns precisely with the claw’s centerline. The linear range of this adjustment depends primarily on the claw’s top opening distance, *L_d_*. To guarantee smooth feeding into the gripping zone, *L_d_* must exceed the maximum minor axis *a*. This kinematic constraint is expressed as follows (Equation 9):

(9) $$L_{d}=2\text{Δ}L_{d}+L_{p}=2D\sin\;\frac{\theta }{2}+L_{p}\geq a_{\max\;}$$

Furthermore, the orientation-adjustment capacity is affected by both the wedge angle *θ* and the instantaneous pod posture. Here, *α* denotes the instantaneous angle between the peanut major axis and the horizontal *x*-axis in the claw-opening plane. Because the initial posture of a biological pod during feeding is not an independently controlled design factor, *α* is not assigned a fixed numerical value in the following derivation. Instead, it is used as a geometric posture variable for describing the lateral envelope required during orientation adjustment.

For a given *θ* and *α*, the lateral envelope width required by the tilted peanut pod can be expressed as (Equation 10)

(10) $$W_{\text{env}}\left(\theta ,\alpha \right)=\frac{\left|\tan\;\left(\alpha -\frac{\theta }{2}\right)b^{2}\cos\;\alpha -a^{2}\sin\;\alpha \right|}{\sqrt{a^{2}+\tan^{2}\left(\alpha -\frac{\theta }{2}\right)b^{2}}}$$

where $W_{\text{env}}\left(\theta ,\alpha \right)=2\left|x\right|$ denotes the full lateral envelope width required by the tilted pod. To accommodate this envelope, the effective opening distance should satisfy (Equation 11)

(11) $$L_{d}=2D\sin\;\frac{\theta }{2}+L_{p}\geq W_{\text{env}}\left(\theta ,\alpha \right).$$

Therefore, for a given *D*, *θ*, and *α*, the corresponding lower-bound requirement for *L_p_* can be written as

(12) $$L_{p}\geq W_{\text{env}}\left(\theta ,\alpha \right)-2D\sin\;\frac{\theta }{2}.$$

This expression describes the geometric coupling among *D*, *θ*, *L_p_*, and the pod posture *α*. In the subsequent experimental-factor selection, this relationship is used to evaluate whether the orientation-envelope requirement becomes active within the selected *θ*–*L_p_* design window, rather than to impose a fixed posture angle.

Additionally, *L_p_* must be bounded. At a constant *θ*, an excessively wide *L_p_* may leave the pod misaligned post-adjustment. Conversely, an undersized *L_p_* forces deeper compression to fully seat the pod, inducing an excessive rigid constraint effect and inflating the required shelling force. Furthermore, the V-groove depth *D* must be limited; an excessive *D* prevents complete pod compression. Given a shell-kernel clearance *g* and a shell thickness *t*, the shelling stroke *M* should remain below *g* + 2*t*. Consequently, *D* is bounded by (Equation 13):

(13) $$D<\frac{b_{\min\;\;}-g_{\min\;\;}-2t_{\min\;\;}}{2}$$

In practice, the V-groove depth *D* must balance effective gripping with seed-protection requirements. To eliminate the risk of crushing, the design must be anchored on the absolute worst-case biological extreme: the smallest peanut (*b*_min_) with the thinnest shell (*t*_min_) and smallest internal clearance (*g*_min_). The baseline physical boundary that guarantees the protrusion tip approaches, but does not aggressively breach, this most vulnerable seed is strictly defined by $\frac{b_{min\;\;}-g_{min\;\;}-2t_{min\;\;}}{2}$.

However, peanuts are highly heterogeneous biological composites. Setting *D* rigidly to this minimum baseline would compromise the gripping stability for larger pods (*b*_max_). To resolve this mechanical paradox, an engineering morphological tolerance window is introduced. The design utilizes a reliability derating factor *η* to bridge the gap between the maximum biological bounding box and the minimum safety baseline.

The finalized robust engineering design formula is established as:

(14) $$D_{\text{design}}=\underset{\text{Absolute Safety Baseline}}{\underset{︸}{\frac{b_{\min}-g_{\min}-2t_{\min}}{2}}}+\eta \cdot \underset{\text{Morphological Compensation Window}}{\underset{︸}{\left[\frac{\left(g_{\min}+2t_{\min}+b_{\min}\right)-\left(b_{\max}-2t_{\min}-g_{\min}\right)}{2}\right]}}$$

where the first term represents a seed-protection reference depth for the most vulnerable pods; the second term is a morphology-compensation correction scaled by the derating factor *η* to balance seed protection and alignment stability across pod-size variation. This derating was introduced to reduce the risk of over-closure and claw-to-claw interference after shell rupture in small pods.

To provide a conservative engineering reference for the seed-protection boundary, a safety boundary *D*_limit_ was estimated using the lower-bound tendency of the measured pod and clearance parameters. Because the shell-kernel clearance and shell thickness were measured from a limited Group B sample size (*n* = 20), this boundary should be interpreted as a conservative design reference rather than as a rigorous tail-probability guarantee (Equation 15).

(15) $$D_{\text{limit}}=\frac{\left(\mu_{g}-3\sigma_{g}\right)+2\left(\mu_{t}-3\sigma_{t}\right)+\left(\mu_{b}-3\sigma_{b}\right)}{2}$$

Substituting the empirical data from **Table 1** yields a conservative reference value of $D_{\text{limit}}\approx 7.32\text{ mm}$. This value was used only as an engineering screening boundary for reducing the risk of excessive intrusion into the kernel-occupied region, rather than as a statistical guarantee of seed preservation probability.

**Table 1:** Statistical data of peanut pre-test samples.

Measurement parameter	Sample group	Sample size (*n*)	Mean (*µ*)	Standard deviation (*σ*)	Minimum	Maximum
Minor axis *a* (mm)	Group A	80	14.77	1.21	11.83	17.13
Major axis *b* (mm)	Group A	80	17.53	1.27	14.42	21.18
Shell-kernel clearance *g* (mm)	Group B	20	2.84	0.65	1.95	4.54
Shell thickness *t* (mm)	Group B	20	1.25	0.41	0.53	2.07

Substituting the conservative engineering tolerance η=0.85 and the empirical boundary values from **Table 1** into Equation 14 yields a finalized depth of $D_{\text{fix}}=5.39\text{ mm}$.

Thus, $D_{\text{fix}}\;\left(5.39\text{ mm}\right)$ is substantially lower than the conservative reference boundary $D_{\text{limit}}\left(7.32\text{ mm}\right)$. This margin supports the selection of η=0.85 and indicates that the fixed depth was designed to remain within a cautious seed-protection range while maintaining stable alignment for variable pod dimensions. However, this result should be interpreted as a conservative engineering safety margin rather than as a statistical guarantee that rigid crushing is completely eliminated.

This analysis demonstrates that the nonlinear effect on *K*_shell_ stems from the coupled interaction between *θ* and *L_p_*, rather than a single parameter. Consequently, *θ* and *L_p_* non-monotonically influence the lateral compression force *F_x_*. Because the depth *D* also depends on the pod dimension *b* and *L_p_*, the optimal values for *θ* and *L_p_* must be determined experimentally.

#### 2.2.2 Geometric constraints and mechanical design of the stress-concentration protrusion

As illustrated in **Figure 8**, Stages 3 and 4 achieve local stress concentration and rupture guidance via the sharp protrusion. The design of its apex angle *γ* and protrusion height *H_t_* must account for multiple mechanical effects.

The protrusion height *H_t_* serves a dual mechanical role. First, it dictates contact dominance: a larger *H_t_* ensures the tip makes initial contact ( $F_{s}\approx F_{s,\text{tip}}$), whereas a smaller *H_t_* causes the flat surface and tip to contact simultaneously, sharing the total force *F_s_*. Second, it is governed by seed safety constraints: *H_t_* must be strictly limited by the shell-kernel clearance *g*. An excessive *H_t_* would cause the protrusion to breach this clearance upon shell rupture, damaging the seed. Therefore, to prevent kernel crushing, *H_t_* must satisfy the following condition (Equation 16):

(16) $$H_{t}\leq \frac{g}{2}+t$$

Moreover, the flat surface width *L_p_* must satisfy the geometric constraints for mounting the sharp protrusion. Given a protrusion height *H_t_* and an apex angle *γ*, the minimum theoretical basal width *W_t_* of the protrusion is $2H_{t}\tan\;(\gamma /2)$. To ensure the protrusion fits entirely on the flat surface, the following condition must hold (Equations 17, 18):

(17) $$W_{t}=2H_{t}\tan\;\left(\frac{\gamma }{2}\right)$$

(18) $$L_{p}\geq W_{t}$$

The apex angle *γ* also serves a dual mechanical role, governed by the trade-off between stress concentration and the wedge-splitting effect. A smaller *γ* (a sharper profile) yields a higher stress concentration factor *K_t_*, facilitating micro-crack initiation along the ventral suture at a lower total gripping force *F_s_*.

Conversely, a larger *γ* (a blunter profile) favors the wedge-splitting effect. Although *K_t_* decreases, the applied *F_s_* generates a greater lateral force component post-initiation to pry the crack open, driving complete separation along the ventral suture. Thus, an excessively small *γ* permits easy crack initiation but lacks adequate splitting capacity, whereas an excessively large *γ* severely hinders initial crack formation.

### 2.3 Physics-constrained high-adaptability parameter design and experimental validation

This section details the experimental framework used to quantify the nonlinear effect of claw geometry on the critical shelling force *F*_crit_ and kernel damage. To address the severe dimensional variability of actual peanuts and the resulting low SNR, the MC-DORSO framework was introduced to combine constrained experimental sampling, mean-normalized response modeling, and parameter optimization. Within this framework, mechanical analysis was used to define the feasible design space and the design-stage regression basis, whereas the final response surface model was determined after experimentation using adaptive stepwise regression. Therefore, the final model is interpreted as a mechanism-guided and data-supported reduced response surface, rather than as a fixed physics-prescribed sparse model.

To minimize *F*_crit_ and evaluate the system’s sensitivity to representative size fluctuations of the tested peanut population, the experiment investigates two variable categories: design control factors and uncontrollable sources of variation (i.e., material dimensions).

As established theoretically, the V-groove depth *D* is a geometric quantity coupled with the wedge opening and pod accommodation process. However, after the seed-protection and morphology-compensation analysis, the V-groove depth was fixed as *D*_fix_ = 5.39 mm for all experimental claw prototypes. Therefore, the experiment focused on four independent control factors (Equation 19):

(19) $$G_{c}=\left\{\theta ,L_{p},\gamma ,H_{t}\right\}$$

Intrinsic dimensional fluctuations in the material act as an objective disturbance that significantly affects the critical shelling force *F*_crit_. Even within a single variety, the three-dimensional geometric dimensions *G*(*a*, *b*) exhibit inherent biological variability. Such random fluctuations primarily cause the unstable mechanical responses and kernel damage observed in traditional equipment. Consequently, this study introduces the pod dimension *G* into the experimental design as an uncontrollable noise factor. The objective is not to optimize the pod size, but to represent the variable processing conditions of agricultural biological materials. This approach ensures a reliable evaluation of the adaptability and stability of the claw’s control parameters *G_c_* across varying sizes, despite high background noise.

To capture the previously identified nonlinear effects, the parameters *L_p_*, *γ*, and *H_t_* are each assigned three levels (L1, L2, L3) to represent distinct physical states around their critical inflection points. Conversely, because the wedge angle *θ* exhibits a monotonic relationship with the shelling force, a two-level design is employed for comparison. To ensure scientific rigor, qualitative descriptors (e.g., “large/small,” “deep/shallow”) for these levels were quantitatively defined.

Prior to the main experiment, a pre-test was conducted to quantify the statistical characteristics of the noise factors and the safety limits of the control factors. Using “Luhua 11” peanuts, 100 samples were selected and divided into Group A (80 samples) and Group B (20 samples). Group A was used to establish the statistical distribution of the dimensional noise factor *G*(*a*, *b*). Group B underwent destructive testing to determine the safety upper bound for the protrusion height *H_t_* by identifying the global minimum shell-kernel clearance *g*_min_.

The measurement data are shown in **Table 1**.

**Figure 10:**
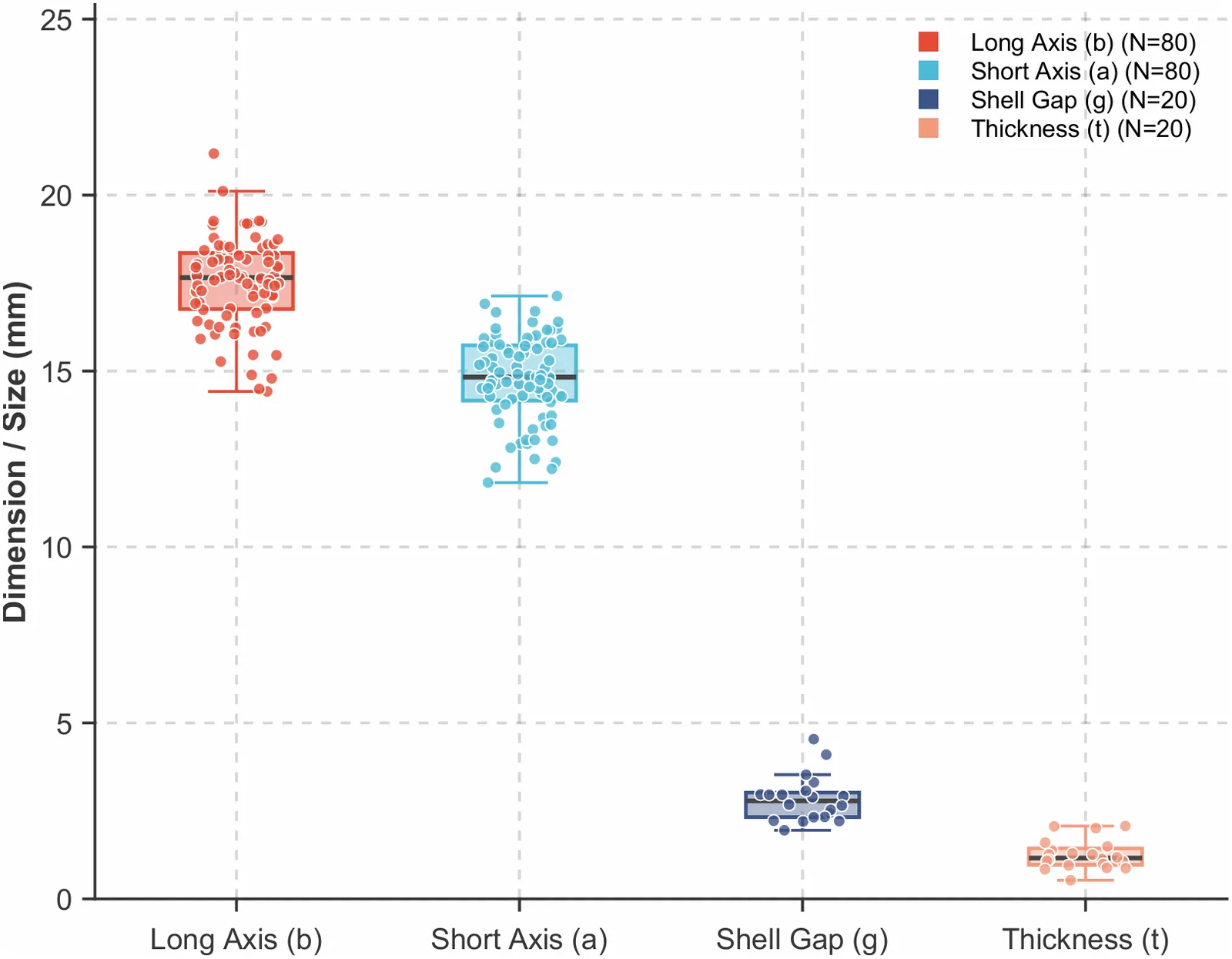
Boxplots of data distributions.

To visually illustrate the fluctuation ranges of the peanut dimensions, boxplots of the data distributions for *a*, *b*, *g*, and *t* were generated, as shown in **Figure 10**.

Based on the pre-test results, establishing the experimental factor levels requires strict adherence to the previously derived mechanical models and boundary conditions, ensuring the design effectively captures critical mechanical states.

First, the three levels for the noise factor *G*(*a*, *b*) (pod dimensions) were defined using the statistical properties of the pre-test samples listed in **Table 1**. The purpose of this classification was not to cover every possible extreme pod size, but to construct statistically distinguishable and experimentally repeatable representative size levels for evaluating the influence of dimensional variability on shelling performance.

The pre-test results showed that both the minor axis *a* and major axis *b* of the tested peanut population approximately followed normal distributions. Therefore, the representative dimensional fluctuation range was defined using the mean and standard deviation of both axes. Within the range of approximately *μ* ± σ, three controlled noise-factor levels were constructed: small, medium, and large.

To guarantee statistical distinction between noise levels while minimizing intra-group dispersion, the three discrete level sets (*L*_1_, *L*_2_, and *L*_3_) for *G* were defined as follows:

(20) $$G\left(a,\;b\right)=\{\begin{array}{ll} L_{1}\left(\text{Small}\right): & \left\{\left(a,b\right)\;|\;\begin{array}{l} \mu_{a}-\sigma_{a}\leq a<\mu_{a}-0.5\sigma_{a},\\ \mu_{b}-\sigma_{b}\leq b<\mu_{b}-0.5\sigma_{b} \end{array}\right\}\\ L_{2}\left(\text{Medium}\right):\; & \left\{\left(a,b\right)\;|\;\begin{array}{l} \mu_{a}-0.5\sigma_{a}\leq a\leq \mu_{a}+0.5\sigma_{a},\\ \mu_{b}-0.5\sigma_{b}\leq b\leq \mu_{b}+0.5\sigma_{b} \end{array}\right\}\\ L_{3}\left(\text{Large}\right): & \left\{\left(a,b\right)\;|\;\begin{array}{l} \mu_{a}+0.5\sigma_{a}<a\leq \mu_{a}+\sigma_{a},\\ \mu_{b}+0.5\sigma_{b}<b\leq \mu_{b}+\sigma_{b} \end{array}\right\} \end{array}$$

Substituting $\mu_{a}=14.77\text{ mm}$, $\sigma_{a}=1.21\text{ mm}$, $\mu_{b}=17.53\text{ mm}$, and $\sigma_{b}=1.27\text{ mm}$ into Equation 20, the actual screening intervals are obtained as follows (Equation 21):

Here, *L*_1_, *L*_2_, and *L*_3_ denote the three representative pod-size levels for the noise factor *G*(*a*, *b*). They should be distinguished from the low, middle, and high control-factor levels listed in **Table 2**.

**Table 2:** Experimental factors and search space boundaries.

Factor	Symbol	Type	Low level (L1)	Midpoint (L2)	High level (L3)
Peanut dimension	*G*	Noise (Discrete)	Small (*L*_1_)	Medium (*L*_2_)	Large (*L*_3_)
Wedge angle	*θ*(*X*_1_)	Control (2-level)	90	–	120
Flat width	*L_p_*(*X*_2_)	Control (Continuous)	10.0 mm	13.0 mm	16.0 mm
Apex angle	*γ*(*X*_3_)	Control (Continuous)	45	60	75
Protrusion height	*H_t_*(*X*_4_)	Control (Continuous)	0.6 mm	1.0 mm	1.4 mm

(21) $$G\left(a,\;b\right)=\{\begin{array}{ll} L_{1}\left(\text{Small}\right): & \left\{\left(a,b\right)\;|\;\begin{array}{l} 13.56\text{ mm}\leq a<14.17\text{ mm},\\ 16.26\text{ mm}\leq b<16.90\text{ mm} \end{array}\right\}\\ L_{2}\left(\text{Medium}\right): & \left\{\left(a,b\right)\;|\;\begin{array}{l} 14.17\text{ mm}\leq a\leq 15.38\text{ mm},\\ 16.90\text{ mm}\leq b\leq 18.17\text{ mm} \end{array}\right\}\\ L_{3}\left(\text{Large}\right): & \left\{\left(a,b\right)\;|\;\begin{array}{l} 15.38\text{ mm}<a\leq 15.98\text{ mm},\\ 18.17\text{ mm}<b\leq 18.80\text{ mm} \end{array}\right\} \end{array}$$

According to Equation 20, samples whose *a* or *b* values fell outside the defined representative intervals were not assigned to the controlled noise-factor groups used for response surface modeling. These samples were not regarded as abnormal biological outliers. Instead, this treatment was adopted to reduce within-group dispersion and maintain clear separation among the small, medium, and large representative size levels.

Second, the level selection for the control factors must strictly satisfy the theoretical boundary conditions. Notably, the strong geometric coupling between *θ* and *L_p_* necessitates their synergistic evaluation.

Determining the levels for *θ* and *L_p_* employed a feasibility-boundary back-calculation strategy. Because the V-groove depth had been fixed as $D_{\text{fix}}=5.39\text{ mm}$ for all claw prototypes, the *θ*–*L_p_* design window was evaluated by combining the feeding-opening requirement with the orientation-envelope activity check.

The feeding-opening lower bound was calculated as (Equation 22)

(22) $$L_{p,C1}\left(\theta \right)=a_{\max\;\;}-2D_{\text{fix}}\sin\;\frac{\theta }{2}.$$

For the orientation-envelope condition, the posture angle *α* was not fixed. Instead, Equation 12 was evaluated over the non-redundant posture range $\alpha \in \left[0^{{}^{\circ}},90^{{}^{\circ}}\right]$ in the claw-opening plane. Using the representative mean pod dimensions from the pre-test, the corresponding projected lower bound was defined as (Equation 23)

(23) $$L_{p,C2}^{\text{env}}\left(\theta \right)=\underset{\alpha \in \left[0^{{}^{\circ}},90^{{}^{\circ}}\right]}{\max}\left[W_{\text{env}}\left(\theta ,\alpha \right){|}_{a=\mu_{a},b=\mu_{b}}-2D_{\text{fix}}\sin\;\frac{\theta }{2}\right].$$

Thus, the effective lower bound in the *θ*–*L_p_* plane was obtained as (Equation 24)

(24) $$L_{p,\min\;\;}^{\text{eff}}\left(\theta \right)=\max\;\left[0,\;L_{p,C1}\left(\theta \right),\;L_{p,C2}^{\text{env}}\left(\theta \right)\right].$$

**Figure 11:**
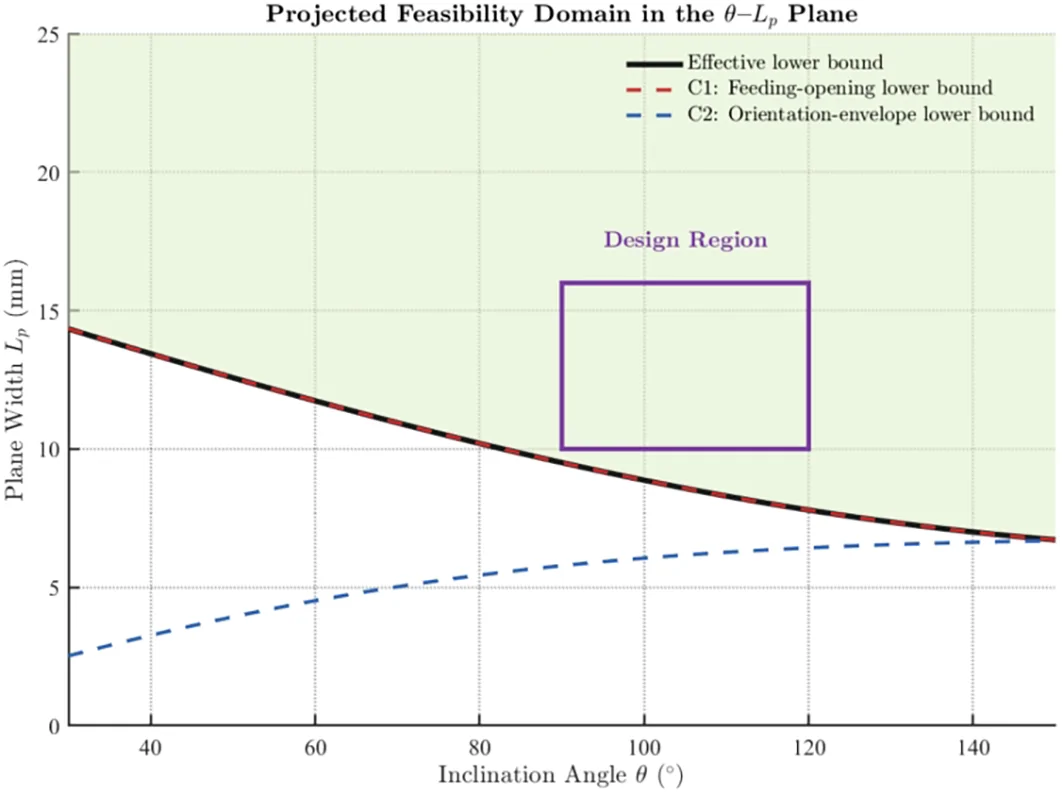
Projected feasibility domain in the *θ*–*L_p_* plane. The red dashed curve represents the feeding-opening lower bound, the blue dashed curve represents the orientation-envelope lower bound after eliminating the posture variable *α*, and the black curve represents the effective lower bound. The purple box indicates the engineering design window used for the constrained D-optimal design.

The resulting projected feasibility map is shown in **Figure 11**. In the selected engineering design window, the orientation-envelope lower bound remained below the practical lower level of *L_p_* = 10 mm and therefore did not further restrict the candidate points. Consequently, the *θ*–*L_p_* experimental window was mainly governed by the feeding-opening boundary and the predefined engineering levels. The lower limit of *θ* was set to 90° by considering the practical minimum flat width of *L_p_* = 10 mm and the feeding-opening boundary. The upper limit of *θ* was set to 120°, where the lateral force $F_{x}\approx 0.29F_{s}$ still maintains basic centering functionality while avoiding excessive lateral restriction. Therefore, the effective experimental ranges were established as $\theta \in \left[90^{{}^{\circ}},120^{{}^{\circ}}\right]$ and $L_{p}\in \left[10,16\right]\text{ mm}$.

Levels for the apex angle *γ* were selected to span the distinct dominant shelling mechanisms. Level L1 (45°) evaluates stress-concentration-dominated scenarios, while Level L3 (75°) emphasizes wedge-splitting-dominated conditions. Level L2 (60°) serves as the transitional intermediate state.

The protrusion height *H_t_* must be bounded by the seed safety constraint defined in the formula derived in the previous text. Based on empirical data from pre-test Group B (minimum shell-kernel clearance $g_{\min\;\;}=1.95\text{ mm}$ and shell thickness $t_{\min\;\;}=0.53\text{ mm}$), the theoretical safety upper bound is $g_{\min\;\;}/2+t_{\min\;\;}\approx 1.5\text{ mm}$. Because a higher *H_t_* within this safe envelope theoretically enhances stress concentration, an equidistant distribution strategy was adopted. Three elevated levels (0.6, 1.0, and 1.4 mm) were established to span the spectrum from a micro-protrusion to the proximity of the safety upper limit, facilitating a comprehensive exploration of potential non-linear optimal values.

### 2.4 Construction of the MC-DORSO framework

#### 2.4.1 Design-stage regression basis and constrained design space definition

The analytical models were used to guide experimental sampling rather than to directly predict *F*_crit_. Hertz contact theory identified *γ* and *H_t_* as variables controlling local contact curvature, whereas the force-decomposition model identified *θ*, *L_p_*, and their interaction as factors governing lateral constraint. Based on these physical couplings, the following design-stage regression basis was used to construct the D-optimal candidate information matrix (Equation 25):

(25) $$Y=\beta_{0}+\underset{\text{Linear main effects}}{\underset{︸}{\sum_{i=1}^{4}\beta_{i}X_{i}}}+\underset{\text{Lateral stiffness coupling term}}{\underset{︸}{\beta_{12}X_{1}X_{2}}}+\underset{\text{Hertzian contact coupling term}}{\underset{︸}{\beta_{34}X_{3}X_{4}}}+\underset{\text{Nonlinear curvature terms}}{\underset{︸}{\beta_{22}X_{2}^{2}+\beta_{33}X_{3}^{2}+\beta_{44}X_{4}^{2}}}+\epsilon$$

This design-stage basis contains ten coefficients including the intercept; therefore, *N*_run_ = 16 was selected to keep the number of runs larger than the number of estimated coefficients while maintaining a manageable experimental workload. The basis was used only for D-optimal sampling and candidate-space construction, whereas the final response surface was subsequently obtained from the experimental data using adaptive stepwise regression. After data acquisition, adaptive stepwise regression was performed on an expanded set of physically plausible terms to obtain the final reduced response surface model.

Under this model, the experimental design reduces to identifying a design matrix X of *N*_run_ =16 test points within a feasibility region Ω that maximizes the determinant of the information matrix $\left|X^{T}X\right|$. Based on the preceding feasibility-boundary analysis, Ω was defined by the feeding-opening constraint, the orientation-envelope check, the seed-protection constraint, and the protrusion-mounting constraint (Equation 26):

(26) $$\Omega =\left\{\left(\theta ,L_{p},\gamma ,H_{t}\right)\in {\mathbb{R}}^{4}|C_{1}\left(\theta ,L_{p}\right)\wedge C_{2}^{\text{env}}\left(\theta ,L_{p}\right)\wedge C_{3}\left(H_{t}\right)\wedge C_{4}\left(L_{p},H_{t},\gamma \right)\right\}.$$

The physical constraints used for candidate-space filtering were defined as follows:

(27) $$\begin{array}{ll} C_{1}: & 2D_{\text{fix}}\sin\;(\theta /2)+L_{p}\geq a_{\max},\\ C_{2}^{\text{env}}: & L_{p}\geq L_{p,C2}^{\text{env}}\left(\theta \right),\\ C_{3}: & H_{t}\leq \frac{g_{min\;\;}}{2}+t_{\min},\\ C_{4}: & L_{p}\geq 2H_{t}\tan\;(\gamma /2). \end{array}$$

where *C*_1_ denotes the feeding-opening constraint, $C_{2}^{\text{env}}$ denotes the orientation-envelope check after eliminating the uncontrolled posture variable *α*, *C*_3_ denotes the seed-protection constraint, and *C*_4_ denotes the protrusion-mounting constraint. In Equation 27, *D*_fix_ is the fixed V-groove depth, *a*_max_ is the maximum minor-axis dimension observed in the pre-test sample population and was used as a conservative feeding-opening boundary; $L_{p,C2}^{\text{env}}\left(\theta \right)$ is the projected lower bound obtained from the orientation-envelope analysis, and *g*_min_ and *t*_min_ are the minimum shell-kernel clearance and shell thickness obtained from the pre-test. The term $2H_{t}\tan\left(\gamma /2\right)$ represents the minimum basal width required by the protrusion. The practical roles of these constraints are summarized in **Table 14** in **Appendix B**.

**Figure 12:**
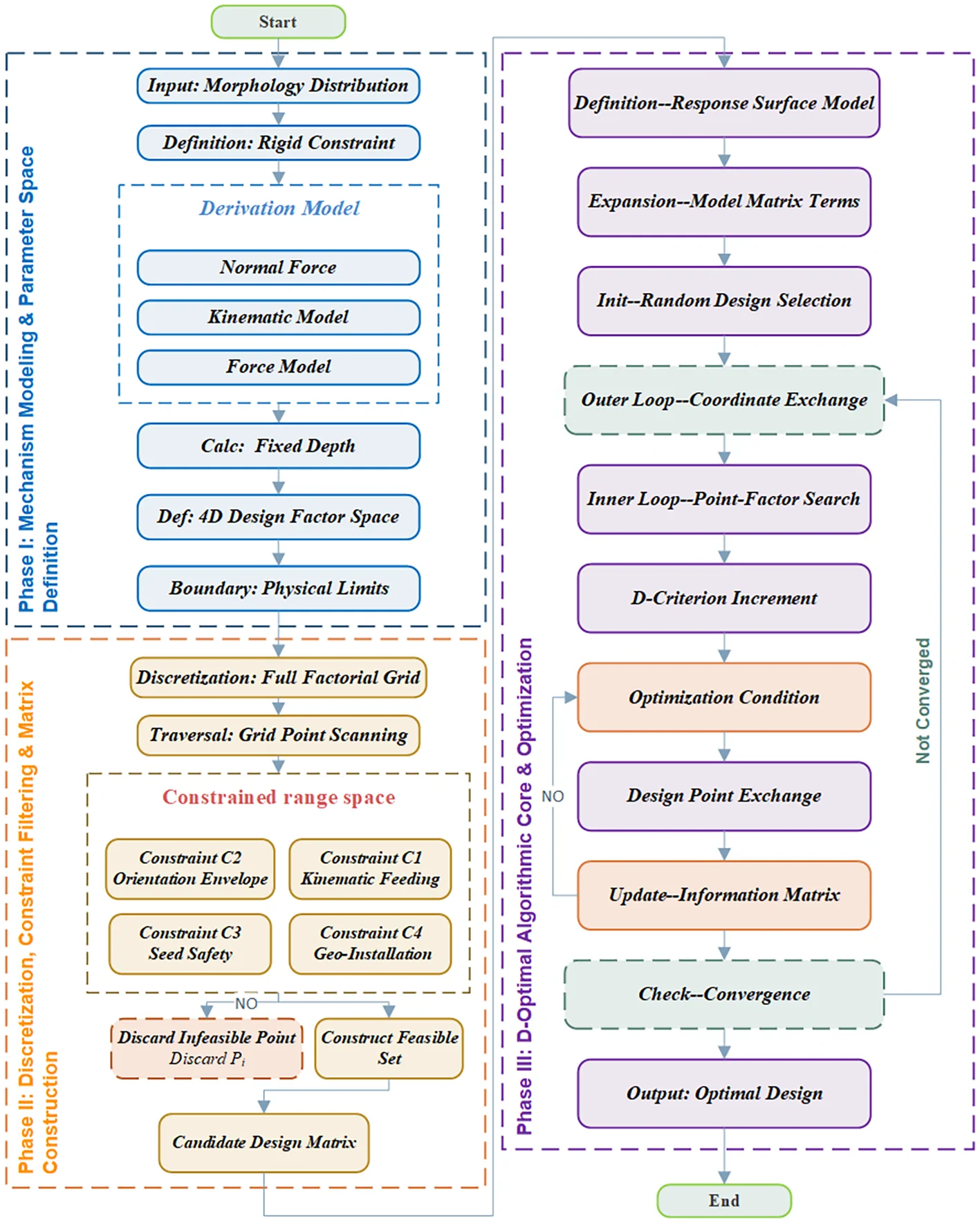
Computational workflow of the mechanism-constrained D-optimal response surface optimization (MC-DORSO) framework.

A constrained candidate-set strategy was used to implement the MC-DORSO framework. As shown in **Figure 12**, the workflow first defines the feasible design space from peanut morphology, mechanical constraints, and factor boundaries. A full discrete candidate set $C_{0}$ is then generated within the ranges of *θ*, *L_p_*, *γ*, and *H_t_*, and Boolean filtering using *C*_1_, $C_{2}^{\text{env}}$, *C*_3_, and *C*_4_ was applied to retain only physically feasible candidates. Here, $C_{2}^{\text{env}}$ was evaluated as an orientation-envelope check after eliminating the uncontrolled posture variable *α*, rather than by assigning a fixed initial posture angle. Within the feasible candidate set C, the coordinate-exchange algorithm selects 16 runs by maximizing the determinant of the information matrix, $\left|X_{\text{D}}^{T}X_{\text{D}}\right|$. The selected design is then used for experimental testing, mean-normalized response modeling, and mixed discrete–continuous parameter optimization. The detailed implementation workflow is provided in **Appendix B**, **Table 13**.

This workflow ensures that D-optimal sampling is performed only within the physically feasible design domain, rather than in an unconstrained mathematical factor space. Therefore, the selected experimental points retain both statistical information content and mechanical feasibility.

Generated by the constrained D-optimal sampling stage of the MC-DORSO framework, the 16 experimental runs are detailed in **Table 3**. The design points are not uniformly distributed; instead, they include several boundary levels of the tested design space, such as *θ* = 90° or 120° and *H_t_* = 0.6 mm or 1.4 mm, as selected by the D-optimality criterion within the pre-screened feasible candidate set.

**Table 3:** 16-run D-optimal experimental design generated via MATLAB.

Run	Wedge angle *θ* (°)	Flat width *L_p_* (mm)	Apex angle γ (°)	Protrusion height *H_t_* (mm)	Fixed depth *D*_fix_ (mm)
1	120.0	10.0	45.0	1.4	5.39
2	90.0	12.1	75.0	0.6	5.39
3	120.0	16.0	45.0	1.4	5.39
4	120.0	10.0	75.0	0.6	5.39
5	120.0	16.0	60.0	0.6	5.39
6	90.0	16.0	75.0	1.4	5.39
7	90.0	10.0	63.0	1.4	5.39
8	90.0	16.0	45.0	1.0	5.39
9	90.0	12.4	45.0	1.4	5.39
10	120.0	10.0	75.0	1.0	5.39
11	120.0	10.0	45.0	0.6	5.39
12	90.0	16.0	75.0	0.6	5.39
13	90.0	10.0	60.0	1.0	5.39
14	90.0	12.7	45.0	0.6	5.39
15	120.0	14.5	75.0	1.0	5.39
16	120.0	13.3	75.0	1.4	5.39

**Figure 13:**
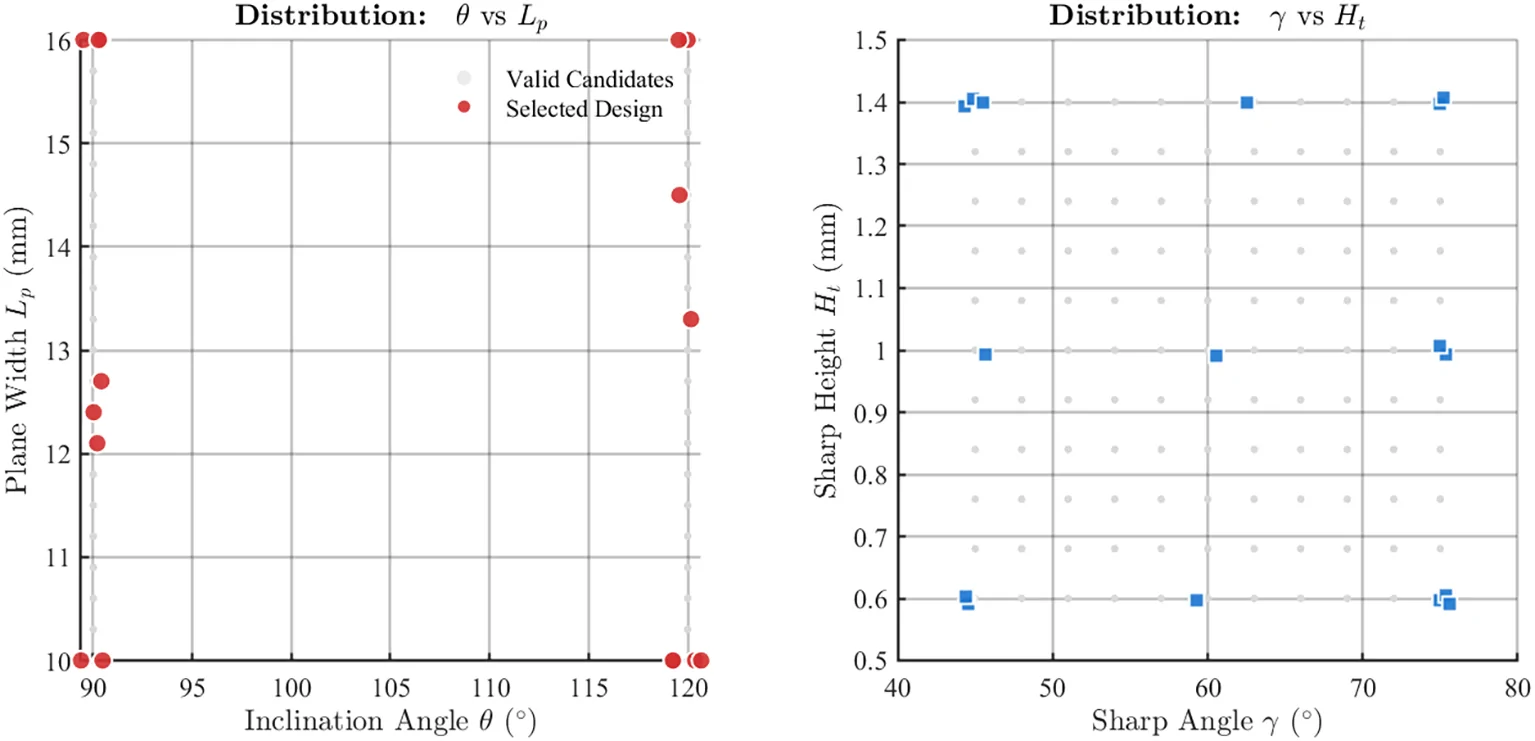
Spatial distribution check of D-optimal experimental points. (Left) Distribution of *θ* and *L_p_* within the projected feasibility window; (Right) Distribution of *γ* and *H_t_* under the seed-protection and protrusion-mounting constraints.

**Figure 13** maps the distribution of the 16 design points across the factor space. The left panel shows that the selected points were located within the pre-screened *θ*–*L_p_* engineering window and satisfied the feeding-opening and orientation-envelope checks. Their final distribution was determined by the D-optimality criterion rather than by uniform spacing.

The right panel shows the distribution of *γ* and *H_t_*, indicating that the selected points covered the protrusion-geometry space under the seed-protection and mounting constraints. The left panel demonstrates that, driven by the D-optimality criterion to maximize predictive power, the points cluster tightly along the effective feasibility boundaries within the highly constrained *θ*–*L_p_* plane. Conversely, the right panel reveals that within the unconstrained *γ*–*H_t_* plane, the design points (blue squares) exhibit excellent space-filling properties, ensuring a robust estimation of both main and interaction effects.

#### 2.4.2 Compression test and response normalization

The 16 shelling claw configurations used in this study were all fabricated using a Bambu Lab A1 3D printer. The printing material was polylactic acid (PLA), with a sparse infill density of 15% and a grid infill pattern. To ensure consistency in the experimental conditions among different configurations, all claw models maintained the same fixed V-groove depth *D*_fix_.

For each control configuration in the inner array (Run *r* = 1,…,16), compression tests were conducted across the three pod-size groups (*k* = 1,2,3) defined in the outer noise array. To mitigate random error, three replicates (*j* = 1,2,3) were performed per size group, totaling nine peanut specimens tested per gripper configuration.

**Figure 14:**
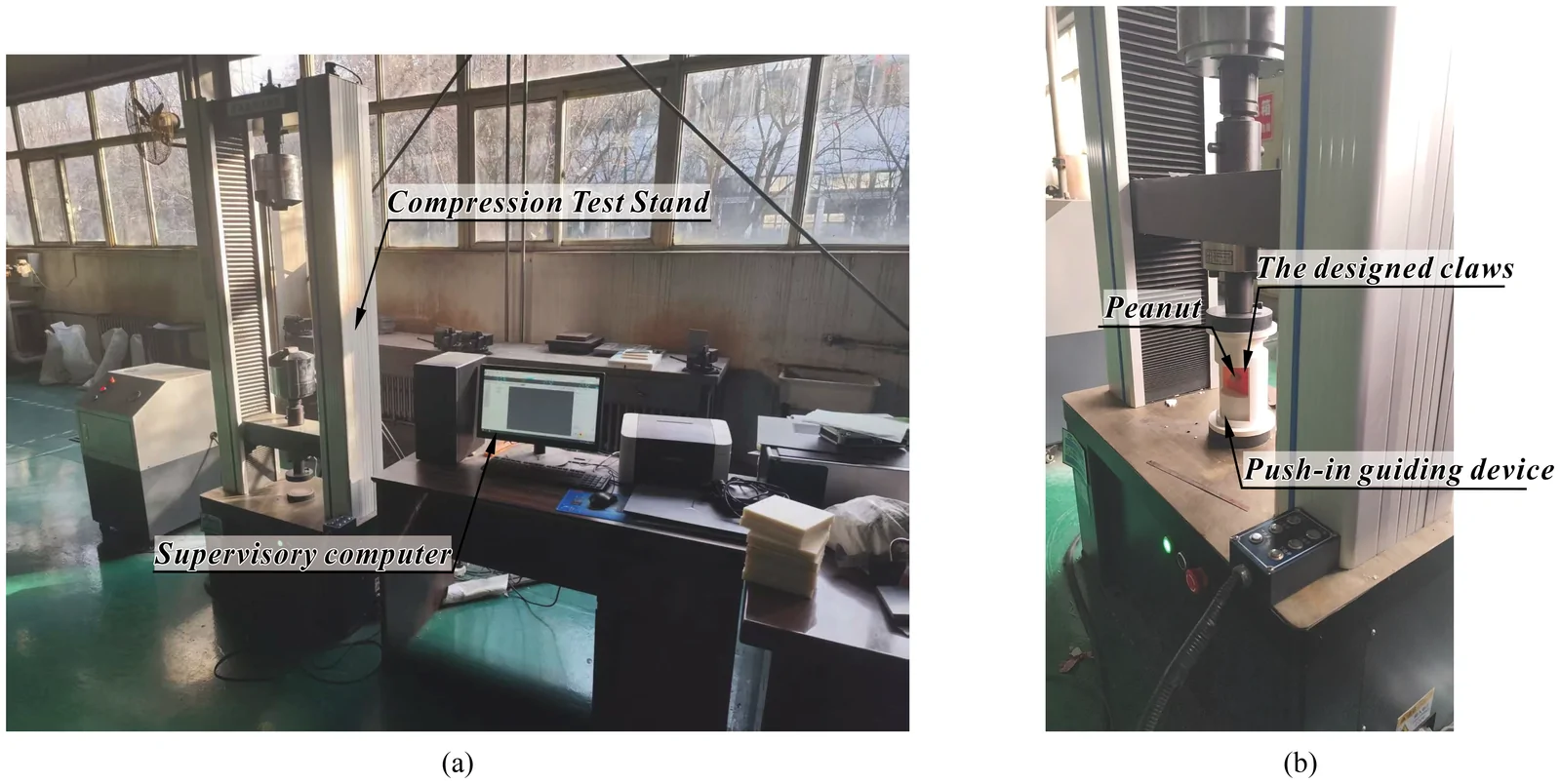
Experimental apparatus: **(a)** stress testing platform and data acquisition system; **(b)** installation details of the flexible shelling claw end effector.

The compression tests were performed using a microcomputer-controlled electronic universal testing machine (Model WDW-100, Jinan Xinguang Testing Machine Manufacturing Co., Ltd., Shandong, China) equipped with a 100 kN load cell. The machine applied a constant displacement rate of 2 mm/min. Real-time force-displacement data were continuously acquired and monitored via the dedicated MaxTest control software (Version 7.73a). In this study, the peak load that resulted in effective macroscopic shell rupture was defined as the critical shelling force, *F*_crit_. The experimental apparatus and claw installation are shown in **Figure 14**.

To reduce statistical bias arising from scale variations in the absolute geometric dimensions of peanuts, this study moves beyond traditional absolute force evaluation and introduces a mean-normalization algorithm. The Relative Shelling Efficiency Index *R_μ_* is defined as the core response indicator (Equation 28):

(28) $$R_{\mu ,rkj}=\frac{F_{\text{crit},rkj}}{{\bar{F}}_{k}}$$

where *F*_crit,_*_rkj_* represents the critical shelling force measured for the *j*-th replicate of the *k*-th pod-size group under the *r*-th claw configuration, and ${\bar{F}}_{k}$ denotes the mean critical shelling force of the *k*-th pod-size group across all configurations and replicates. The physical significance of this index is straightforward: *R_μ_* < 1.0 indicates that the gripper design outperforms the average baseline for that pod size. By applying this dimensionless normalization, the size-dependent baseline shift in shelling force was reduced. This allowed the subsequent regression model to evaluate the relative contribution of claw geometry to shelling efficiency more clearly within the representative size groups.

#### 2.4.3 Prototype fabrication and dimensional tolerance measurement

The purpose of using PLA prototypes in this study was to rapidly fabricate and compare 16 different claw geometries under the same material and manufacturing conditions. Therefore, the experimental results mainly reflect the relative influence of claw geometric parameters on shelling performance. Meanwhile, PLA differs from industrial metallic materials in elastic modulus, surface roughness, and local stiffness. Therefore, the absolute shelling force values obtained in this study should not be directly extrapolated to industrial applications using metallic claws.

For thin-walled curved structures, small geometric or mass-distribution defects can markedly alter the structural response, vibration behavior, and performance consistency, which highlights the necessity of evaluating the robustness of optimized geometries against manufacturing deviations Liu et al. (2025).

**Figure 15:**
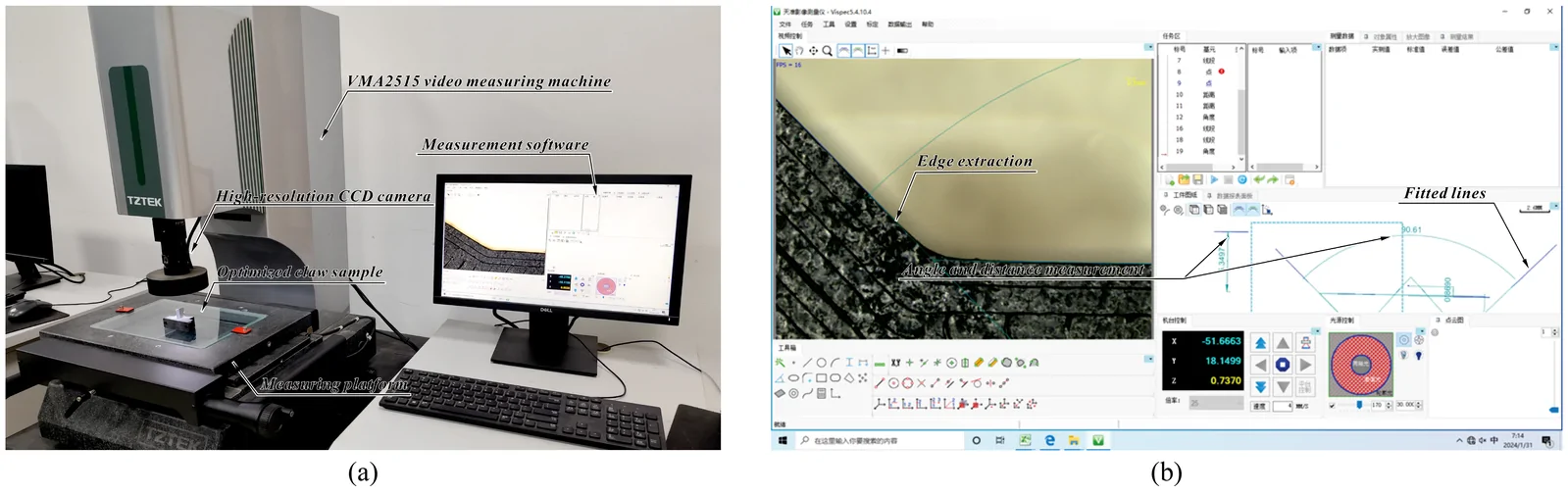
Dimensional inspection for process-level manufacturing deviation evaluation using a VMA2515 video measuring machine: **(a)** measurement setup for the 3D-printed claw sample; **(b)** image-based extraction and fitting of angular and linear geometric features.

Therefore, to quantify process-level dimensional deviations under the same 3D-printing conditions, five samples of a representative printed claw configuration were repeatedly fabricated and measured, as shown in **Figure 15**. The measured angular and linear deviations were used as representative manufacturing tolerances for the subsequent local model-based sensitivity analysis.

The measuring range of the video measuring machine was 250×150×150 mm. The XY-axis measurement accuracy was $\left(3.0+L_{\text{m}}/200\right)\;\mu \text{m}$, where *L*_m_ is the measured length in millimeters, the grating ruler resolution was 1.0 *μ*m, the optical magnification was 0.7–4.5, and the image magnification was 28–184×. During measurement, surface illumination and contour illumination were adjusted according to the local contour features of the claw to obtain clear edge images. The claw boundaries were then extracted in the image measurement software, and the geometric parameters were obtained through line fitting, intersection positioning, and distance calculation. Each sample was measured under the same measurement reference and placement orientation. The measured values were compared with the nominal computer-aided design (CAD) dimensions to calculate the absolute deviations. The measured deviations were used to determine the local manufacturing tolerances and further support the subsequent tolerance sensitivity analysis.

For any geometric parameter *X_k_*, its local manufacturing tolerance was defined as the maximum absolute deviation from the nominal dimension among all repeatedly printed samples (Equations 29, 30):

(29) $${\text{Δ}}_{\text{ang}}=\underset{m=1,\ldots ,5}{\max}\left|A_{m}^{\text{meas}}-A^{\text{nom}}\right|$$

(30) $${\text{Δ}}_{\text{lin}}=\underset{m=1,\ldots ,5}{\max}\left|L_{m}^{\text{meas}}-L^{\text{nom}}\right|$$

where *m* denotes the printed sample index, $A_{m}^{\text{meas}}$ and $L_{m}^{\text{meas}}$ are the measured angular and linear dimensions of the *m*-th printed sample, and *A*^nom^ and *L*^nom^ are the corresponding CAD nominal values. The resulting angular manufacturing deviation Δ_ang_ and linear dimensional manufacturing deviation Δ_lin_ were used as representative manufacturing deviations under the same 3D-printing process for the subsequent local tolerance sensitivity analysis.

### 2.5 Validation test design

Existing commercial peanut shellers are mostly roller–concave continuous systems, in which impact, rubbing, extrusion, tearing, screening, and multi-pod interactions occur simultaneously. These machines are suitable for evaluating overall shelling efficiency and batch-processing performance, but their working processes cannot be equivalently reproduced on the single-pod quasi-static compression platform used in this study. Therefore, to isolate the contribution of the stress-concentration claw under identical loading conditions, flat-press compression was used as the mechanical control. This control represents whole-pod compression without a local stress-concentration structure and provides a direct and reproducible baseline for comparing the force-reduction and kernel-preservation effects of the optimized claw.

In the validation test, 60 peanut pods of the same “Luhua 11” batch were randomly selected to evaluate the actual force-reduction performance of the optimized claw under random size fluctuations. All samples were thoroughly mixed before testing and then randomly divided into the flat-press control group and the optimized claw group, with 30 pods in each group. The two groups were tested using the same compression speed, testing platform, and criterion for identifying the critical shelling force.

### 2.6 Evaluation of kernel damage and biological viability

To further verify the low-damage characteristics achieved by the final optimized claw, an additional post-shelling kernel damage and biological viability evaluation was conducted. The “Luhua 11” peanut pods were used as the test material. A total of 100 pods were randomly selected, thoroughly mixed, and then randomly divided into the flat-press control group and the optimized claw group, with 50 pods in each group. Subsequently, constant-speed compression was performed at 2 mm/min, and loading was stopped immediately once initial macroscopic shell rupture occurred.

**Figure 16:**
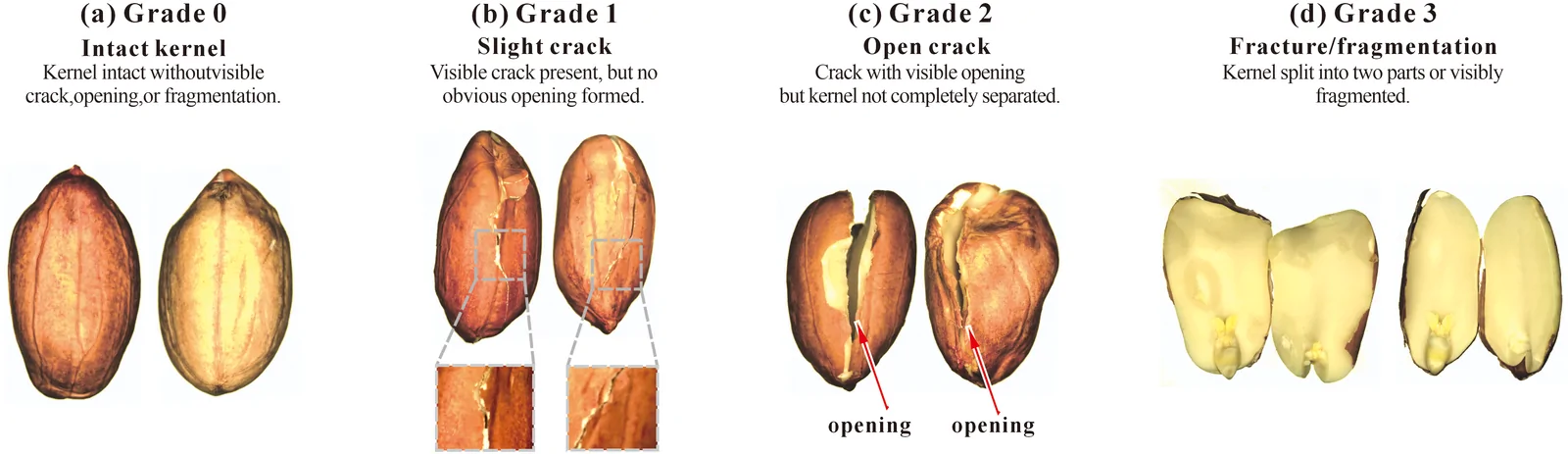
Visual classification criteria for macroscopic mechanical damage of peanut kernels. **(a)** Grade 0: intact kernel without visible damage; **(b)** Grade 1: slight crack without obvious opening; **(c)** Grade 2: open crack without complete separation; **(d)** Grade 3: fracture or fragmentation.

After shell rupture, the target kernels obtained from the loaded region of the two groups were collected and evaluated for macroscopic mechanical damage. Considering that kernel damage after flat-press treatment mainly appeared as cotyledon cracks, crack opening, or complete fracture rather than simple seed-coat peeling, visible mechanical damage grades were established according to the apparent cracking severity of the kernels. Four grades were defined: Grade 0 indicated no visible damage, with an intact kernel appearance and no obvious cracks, openings, or fragmentation; Grade 1 indicated slight cracking, where surface cracks were visible but no obvious crack opening was formed and the kernel remained structurally intact; Grade 2 indicated open cracking, where the crack was relatively large and visibly opened, but the kernel was not completely fractured or separated; and Grade 3 indicated fracture or fragmentation, where the kernel had split into two halves or showed obvious fragmentation. Representative appearances of each damage grade are shown in **Figure 16**.

Based on the number of kernels in each damage grade, the intact-kernel rate, visible mechanical damage rate, and severe mechanical damage rate were further calculated. Grades 1–3 were counted as visible mechanical damage, whereas Grades 2–3 were counted as severe mechanical damage. The calculation formulas are as follows (Equations 31–33):

(31) $$P_{\text{intact}}=\frac{D_{0}}{N_{t}}\times 100\%$$

(32) $$P_{\text{visible}}=\frac{D_{1}+D_{2}+D_{3}}{N_{t}}\times 100\%$$

(33) $$P_{\text{severe}}=\frac{D_{2}+D_{3}}{N_{t}}\times 100\%$$

where *N_t_* denotes the total number of recovered target kernels that could be evaluated after shell rupture; *D*_0_, *D*_1_, *D*_2_, and *D*_3_ denote the numbers of kernels classified as Grade 0, Grade 1, Grade 2, and Grade 3 damage, respectively; P_intact_ denotes the intact-kernel rate, P_visible_ denotes the visible mechanical damage rate, and *P*_severe_ denotes the severe mechanical damage rate.

Subsequently, to further evaluate the effect of the shelling process on seed biological viability, a between-paper germination test was conducted for kernels from different treatment groups. After mechanical treatment, seed-coat integrity and visible mechanical damage were first recorded. Then, kernels from each treatment group were surface-disinfected using the same procedure: soaking in 1% sodium hypochlorite solution for 10 min, thoroughly rinsing with sterile water, and removing surface moisture with sterile filter paper. The treated kernels were then placed between moistened germination papers and incubated in a temperature-controlled chamber.

The germination test used all kernels recovered after shell rupture from each treatment group as the statistical population. The incubation temperature was set to 25 ± 1 °C, and the kernels were incubated in darkness. On the 5th day of incubation, the number of normally germinated kernels was recorded. Kernels with a radicle visibly protruding through the seed coat and showing normal early seedling development were classified as normally germinated, whereas kernels that failed to develop into normal seedlings were classified as non-normally germinated.

The germination rate *G_r_* was calculated as follows (Equation 34):

(34) $$G_{r}=\frac{N_{\text{germ}}}{N_{t}}\times 100\%$$

where *N*_germ_ denotes the number of normally germinated kernels on the 5th day of incubation, and *N_t_* denotes the number of kernels recovered from the loaded region after shell rupture in the corresponding treatment group.

By comprehensively evaluating the visible mechanical damage rate, severe mechanical damage rate, and germination rate, it is possible to more directly determine whether the optimized claw improves kernel preservation while reducing the critical shelling force.

## 3 Results

### 3.1 Response surface modeling and interaction mechanism analysis

**Figure 17:**
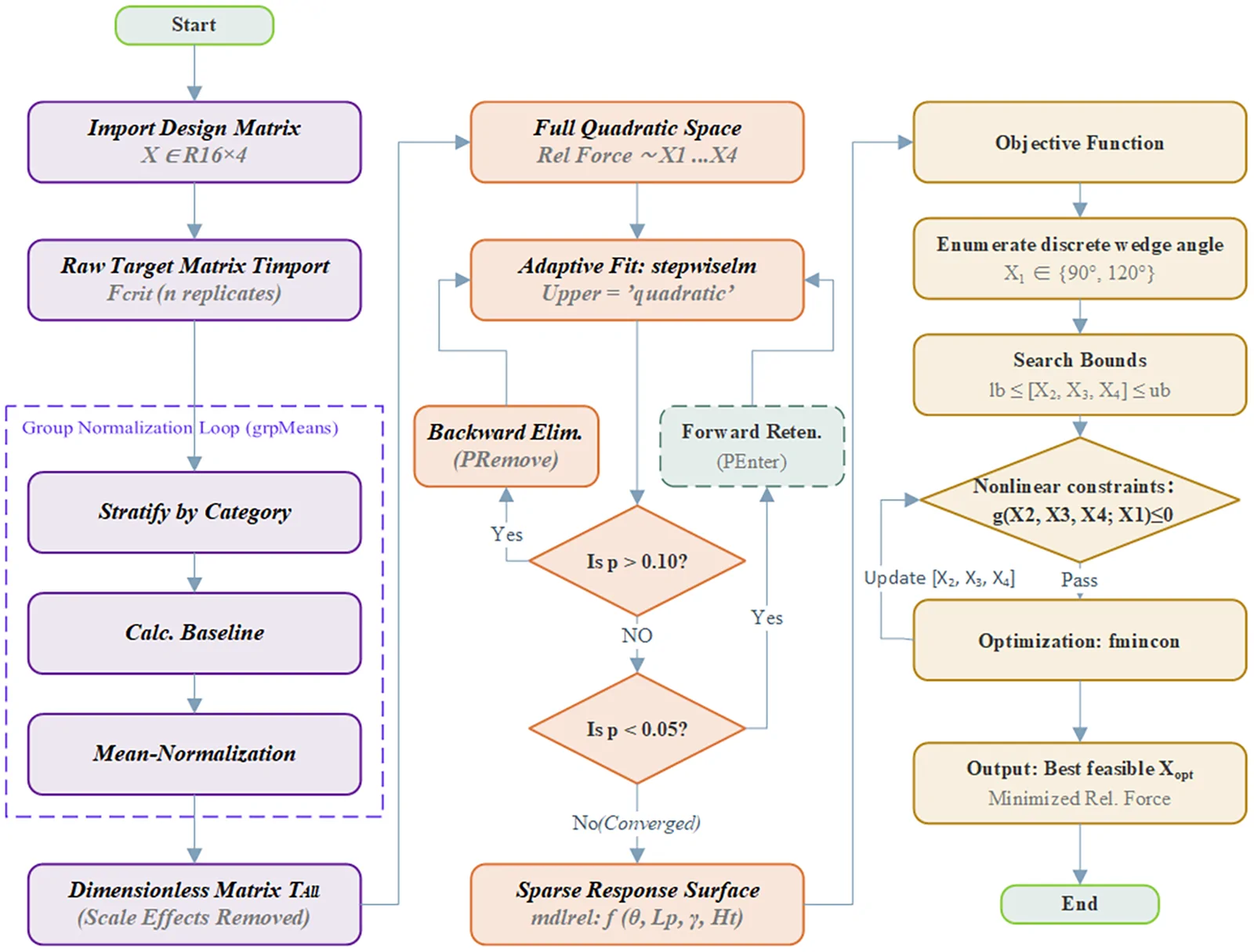
Data-analysis and parameter-optimization architecture within the MC-DORSO framework.

To systematically process highly variable biological data and isolate the geometry-related mechanical effects of the shelling claw, this study developed a multi-phase computational pipeline. As illustrated in **Figure 17**, this pipeline represents the data-analysis and optimization stage of the MC-DORSO framework, integrating mean normalization, mechanism-guided candidate modeling, adaptive stepwise regression, and mixed discrete–continuous constrained optimization. This algorithmic framework ensures that subsequent statistical analyses and optimal parameter derivations remain less sensitive to inherent material fluctuations.

#### 3.1.1 Regression model development and analysis of variance

Following the completion of all 144 shelling trials, the response metrics for each design configuration were statistically aggregated. The compiled experimental data are detailed in **Table 4**.

**Table 4:** Response surface design scheme and experimental shelling efficiency record based on stepwise regression.

Run	Control factors				Response metrics	
	*θ*(*X*_1_) [°]	*L_p_*(*X*_2_) [mm]	*γ*(*X*_3_) [°]	*H_t_*(*X*_4_) [mm]	Measured mean $\bar{F}$ [N]	Relative index *R_μ_*
1	120.0	10.0	45.0	1.4	69.44	1.0114
2	90.0	12.1	75.0	0.6	67.85	0.9892
3	120.0	16.0	45.0	1.4	82.58	1.2091
4	120.0	10.0	75.0	0.6	53.54	0.7831
5	120.0	16.0	60.0	0.6	59.58	0.8593
6	90.0	16.0	75.0	1.4	64.96	0.9439
7	90.0	10.0	63.0	1.4	66.09	0.9658
8	90.0	16.0	45.0	1.0	63.31	0.9437
9	90.0	12.4	45.0	1.4	70.84	1.0272
10	120.0	10.0	75.0	1.0	57.96	0.8556
11	120.0	10.0	45.0	0.6	74.18	1.0761
12	90.0	16.0	75.0	0.6	67.82	0.9938
13	90.0	10.0	60.0	1.0	93.26	1.3624
14	90.0	12.7	45.0	0.6	63.95	0.9333
15	120.0	14.5	75.0	1.0	62.24	0.9133
16	120.0	13.3	75.0	1.4	77.39	1.1327

The measured mean represents the arithmetic mean of all sample forces within a given run; the relative index *R_μ_* is the cross-size normalized efficiency coefficient calculated according to the specified formula. The table reports run-level summary values averaged across the three representative pod-size groups.

Prior to response surface modeling, a multi-factor analysis of variance (ANOVA) was performed on all 144 raw experimental trials to validate the experimental design and response metrics. The mechanical configurations and pod dimensions were designated as independent variables, with the critical shelling force *F*_crit_ serving as the response. The findings are summarized in **Table 5**.

**Table 5:** Multi-factor ANOVA results for the 144 raw experimental samples.

Source of variation	Sum of squares (SS)	Degrees of freedom (df)	Mean square (MS)	*F* value	*P* value
Mechanical Configuration	13045.5	15	869.70	3.87	< 0.001^∗∗^
Peanut Size	14011.9	2	7005.94	31.15	< 0.001^∗∗^
Interaction (Config × Size)	4253.0	30	141.77	0.63	0.9246^(ns)^
Residual Error	21591.3	96	224.91	–	–
Total	52901.6	143	–	–	–

**indicates high significance (*P* < 0.01); *ns* indicates non-significance.

As shown in **Table 5**, both peanut size and mechanical configuration had significant effects on the absolute critical shelling force, whereas their interaction was not significant. This indicates that peanut size mainly produced a baseline shift in shelling force, while the relative trends among mechanical configurations remained broadly consistent across size groups.

**Figure 18:**
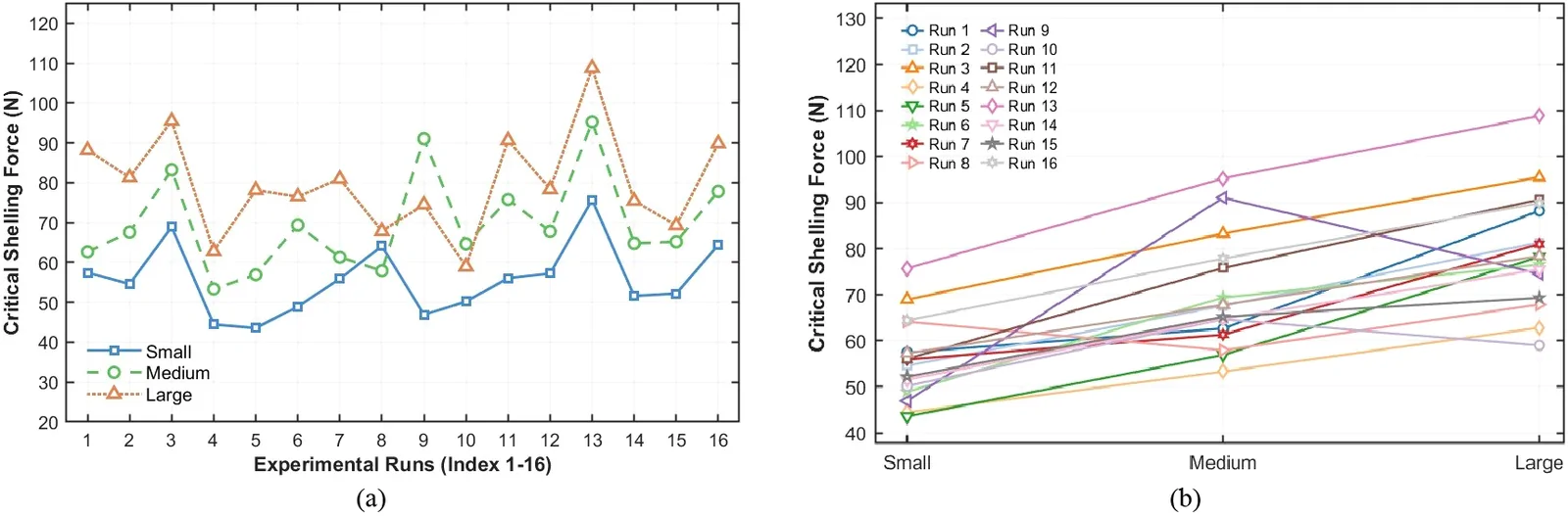
Interaction effect analysis between mechanical configurations and representative peanut pod-size groups on the critical shelling force: **(a)** force variation of the small, medium, and large pod-size groups across 16 experimental runs; **(b)** interaction trajectories between mechanical configurations and pod-size groups. The relatively parallel trajectories are consistent with the statistically non-significant interaction between mechanical configuration and pod-size group.

**Figure 18** further shows this pattern: the three size groups were vertically separated, but their variation trends across the 16 configurations were generally similar. Therefore, the mean-normalized index *R_μ_* was adopted before response surface modeling to reduce size-dependent baseline effects. To reduce random individual defects and avoid overfitting, the 144 raw trials were aggregated into *N*_state_ = 48 physical states, corresponding to 16 configurations and three representative size groups.

As shown in **Table 6**, the significant terms indicate that protrusion height *H_t_* affected the relative shelling response both as a main effect and through its interactions with wedge angle and flat width, as reflected by *X*_4_, *X*_1_*X*_4_, and *X*_2_*X*_4_. In addition, the significant *X*_2_*X*_3_ interaction and $X_{3}^{2}$ term suggest that the coupling between flat width and apex angle, as well as the nonlinear effect of apex angle, also contributed to the response variation.

**Table 6:** ANOVA for the regression model of relative shelling efficiency (*R_μ_*).

Source	SS	df	MS	F-statistic	*P*-value	Sig.
Model	1.1616	8	0.1452	10.68	< 0.0001	**
*X*_1_-*θ*	1.14 × 10^−2^	1	1.14 × 10^−2^	0.84	0.3659	ns
*X*_2_-*L_p_*	8.98 × 10^−3^	1	8.98 × 10^−3^	0.66	0.4221	ns
*X*_3_-*γ*	4.99 × 10^−2^	1	4.99 × 10^−2^	3.67	0.0628	ns
*X*_4_-*H_t_*	8.38 × 10^−2^	1	8.38 × 10^−2^	6.16	0.0175	*
*X*_2_ *X*_3_	8.71 × 10^−2^	1	8.71 × 10^−2^	6.41	0.0155	*
*X*_1_ *X*_4_	3.41 × 10^−1^	1	3.41 × 10^−1^	25.06	< 0.0001	**
*X*_2_ *X*_4_	2.63 × 10^−1^	1	2.63 × 10^−1^	19.34	< 0.0001	**
$X_{3}^{2}$	3.17 × 10^−1^	1	3.17 × 10^−1^	23.31	< 0.0001	**
Residual Error	5.30 × 10^−1^	39	1.36 × 10^−2^	–	–	–
Total	1.69	47	–	–	–	–

SS, sum of squares; df, degrees of freedom; MS, mean square; Sig., significance. **indicates high significance (*P* < 0.01); * indicates significance (*P* < 0.05); *ns* indicates non-significance.

Although the main effects of *X*_1_, *X*_2_, and *X*_3_ were not individually significant, they were retained according to the hierarchy principle because they were associated with significant interaction or quadratic terms Morris et al. (2023). Considering the biological variability and random rupture behavior of peanut pods, the final model was used as an empirical guidance tool for constrained parameter optimization rather than as a deterministic predictor of individual pod rupture. Therefore, the final reduced model was interpreted as an empirical response surface fitted on the constrained D-optimal design, rather than as a deterministic physics-prescribed model. The final second-order response surface regression model is shown in Equation 35:

(35) $$\text{Rel}\_\text{Force}\left(R_{\mu }\right)=1.39696292-0.01997845X_{1}-0.19946395X_{2}+0.13063843X_{3}-3.30489789X_{4}+0.00138918X_{2}\cdot X_{3}+0.01904888X_{1}\cdot X_{4}+0.11081805X_{2}\cdot X_{4}-0.00125977X_{3}^{2}$$

#### 3.1.2 Regression model diagnostic analysis

**Figure 19:**
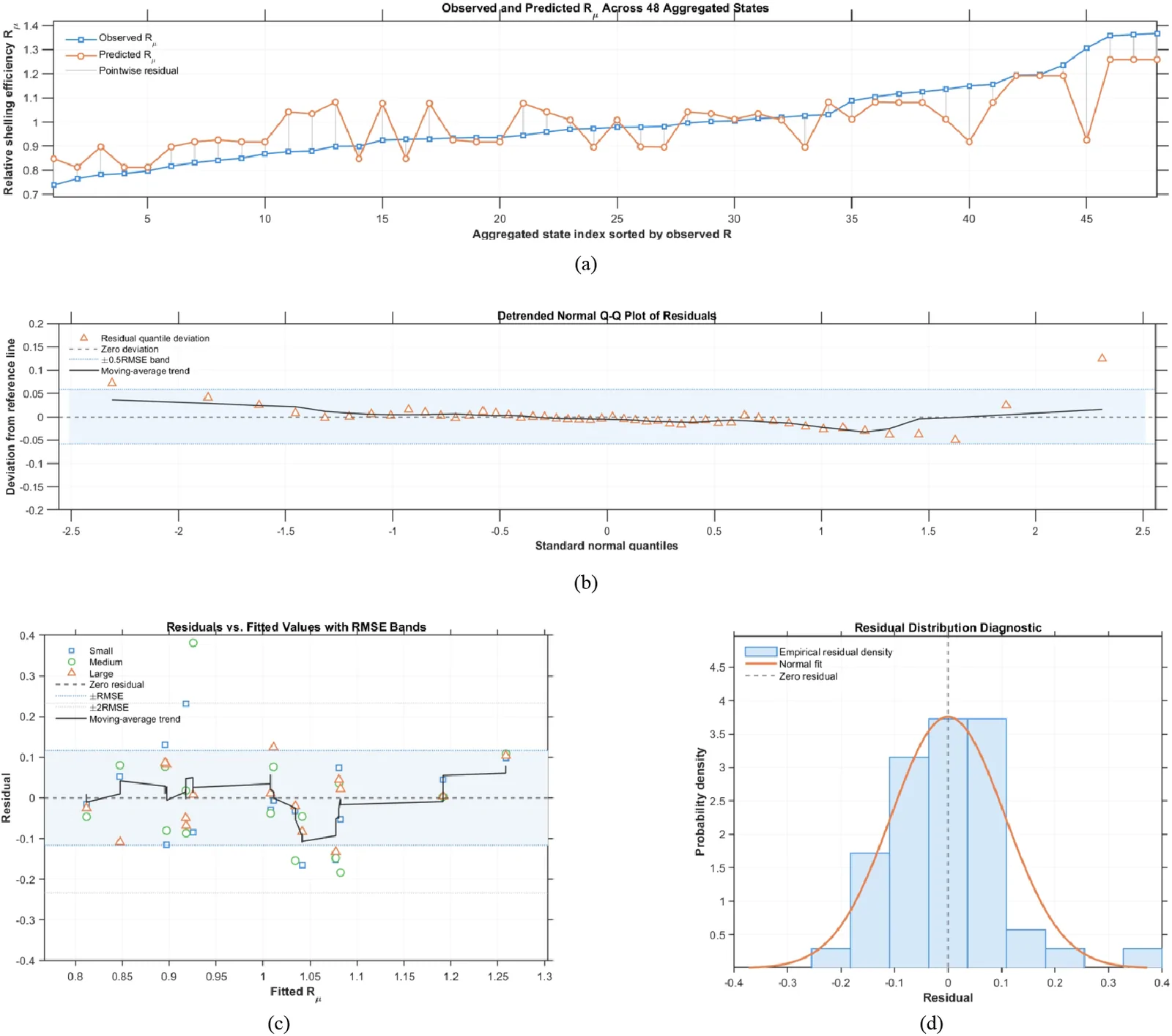
Regression diagnostic plots for the reduced response surface model: **(a)** observed and predicted *R_μ_* across the 48 aggregated physical states; **(b)** detrended normal Q-Q plot of residuals; **(c)** residuals versus fitted values with root mean square error (RMSE)-based reference bands; **(d)** residual distribution diagnostic with empirical density and normal-fit curve.

To further improve the transparency of the regression analysis, regression diagnostics were performed on the final model using the 48 aggregated physical states formed by 16 mechanical configurations and three representative size groups. In this study, the 144 raw compression trials were mainly used for multi-factor ANOVA to examine the effects of mechanical configuration, peanut size group, and their interaction on the absolute critical shelling force *F*_crit_. In contrast, the final response surface model and its diagnostic analysis were based on the 48 aggregated physical states and were used to evaluate the influence of claw geometric parameters on the relative shelling efficiency index *R_μ_*. The diagnostic results are shown in **Figure 19**.

The diagnostic plots in **Figure 19** show that the predicted and observed *R_μ_* values followed generally consistent trends, and the residuals were mainly distributed around zero without obvious systematic bias or severe heteroscedasticity. Although deviations remained in some high-response cases, this is expected for highly heterogeneous biological materials. Therefore, the reduced response surface model was considered adequate for identifying dominant geometry-related trends and guiding constrained parameter optimization.

### 3.2 Constrained parameter optimization and engineering validation

#### 3.2.1 Global parameter optimization based on relative efficiency

In the parameter-optimization stage of the MC-DORSO framework, the objective shifts from conventional multi-objective trade-offs to prioritizing force-reduction performance within the tested representative dimensional range. Specifically, subject to geometric interference and seed safety constraints, the goal is to identify the optimal parameter configuration, *G*_c,opt_, that minimizes the global relative shelling efficiency index, *R_μ_*.

The optimization problem is formulated as the following single-objective minimization task (Equation 36):

(36) $$\{\begin{array}{l} \min\;R_{\mu }\left(X_{1},X_{2},X_{3},X_{4}\right)\\ \text{s}.\text{t}.\;C_{1}\left(X_{1},X_{2}\right),\;C_{2}^{\text{env}}\left(X_{1},X_{2}\right),\;C_{3}\left(X_{4}\right),\;C_{4}\left(X_{2},X_{4},X_{3}\right),\\ \;X_{1}\in \left\{90^{{}^{\circ}},120^{{}^{\circ}}\right\},\\ \;X_{2}\in \left[10,16\right]\;\text{mm},\;X_{3}\in \left[45^{{}^{\circ}},75^{{}^{\circ}}\right],\;X_{4}\in \left[0.6,1.4\right]\;\text{mm}. \end{array}$$

Because the wedge angle *θ* was tested as a two-level engineering factor, it was treated as a discrete candidate variable during optimization rather than as a continuously optimized parameter. Specifically, the optimization was performed by evaluating the two tested wedge-angle levels (90° and 120°), while *L_p_*, *γ*, and *H_t_* were optimized within their feasible ranges under the geometric constraints. The final configuration therefore represents the best-performing parameter combination within the tested mixed discrete–continuous design space.

Therefore, based on the above mixed discrete–continuous optimization, the final optimal parameter combination and its model-predicted response are shown in **Table 7**.

**Table 7:** Optimization results for the optimized force-reduction configuration and model prediction.

Optimal geometric parameters				Model prediction
*θ*[°]	*L_p_*[mm]	*γ*[°]	*H_t_*[mm]	Predicted Index (*R_μ_*)
120.0	16.0	45.0	0.6	0.5885

#### 3.2.2 Local tolerance sensitivity of a representative printed configuration

To further evaluate the influence of 3D-printing-induced manufacturing deviations on the predicted claw response, the sixth claw configuration in the D-optimal design, which had been physically fabricated and tested, was selected as a representative printed configuration for local tolerance sensitivity analysis. Its nominal parameters were *θ* = 90°, *L_p_* = 16 mm, *γ* = 75°, and *H_t_* = 1.4 mm. This configuration combines a relatively large flat width, large apex angle, and high protrusion height, and therefore provides a useful boundary case for evaluating how the V-shaped guiding structure and stress-concentration protrusion respond to manufacturing deviations under boundary geometric conditions.

For $x=\left(X_{1},X_{2},X_{3},X_{4}\right)=\left(\theta ,L_{p},\gamma ,H_{t}\right)$, only one parameter was perturbed at a time while the other three parameters were fixed at their nominal values. To avoid extrapolation beyond the original design space, the local perturbation interval of the *j*-th parameter was truncated by the tested design bounds (Equations 37, 38):

(37) $$X_{j,\text{low}}=\max\;\left(X_{j,\min\;\;},X_{j,0}-{\text{Δ}}_{j}\right)$$

(38) $$X_{j,\text{up}}=\min\;\left(X_{j,\max\;\;},X_{j,0}+{\text{Δ}}_{j}\right)$$

where *X_j_*_,0_ denotes the nominal value of the *j*-th parameter, *X_j_*_,min_ and *X_j_*_,max_ denote the lower and upper bounds of this parameter in the original design space, respectively, and Δ*_j_* denotes the corresponding process-level manufacturing deviation. For the angular parameters *θ* and *γ*, Δ*_j_* = Δ_ang_; for the linear parameters *L_p_* and *H_t_*, Δ*_j_* = Δ_lin_.

For each geometric parameter, a one-factor worst-case perturbation method was used for local tolerance sensitivity analysis. Specifically, only the parameter currently being examined, *X_j_*, was varied while the other three parameters were kept at their nominal values. Since the local feasible tolerance interval had already been determined by *X_j_*_,low_ and *X_j_*_,up_, the lower bound, nominal value, and upper bound of this parameter were used as candidate perturbation points. The candidate perturbation set for the *j*-th parameter was defined as follows (Equation 39):

(39) $$C_{j}=\left\{X_{j,\text{low}},\;X_{j,0},\;X_{j,\text{up}}\right\}$$

The candidate values in $C_{j}$ were then substituted into the response surface model while keeping the other three parameters unchanged. The candidate value that produced the maximum predicted *R_μ_* was defined as the worst-case value of that parameter under one-factor perturbation (Equation 40):

(40) $$X_{j,\text{worst}}=\arg\;\underset{X_{j}\in C_{j}}{\max}\;R_{\mu }\left(X_{1,0},\ldots ,X_{j},\ldots ,X_{4,0}\right)$$

where $C_{j}$ denotes the candidate perturbation set for the *j*-th parameter, and *X_j_*_,worst_ denotes the selected one-factor worst-case perturbation value from this set. Since a larger *R_μ_* indicates a higher relative shelling force and weaker force-reduction performance, this value corresponds to the most unfavorable one-factor perturbation for force-reduction performance within the current process-level tolerance range. The four-parameter combination formed by *X_j_*_,worst_ and the other three nominal parameters was denoted as ***x****_j_*_,worst_.

Since a larger *R_μ_* indicates a higher relative shelling force and weaker force-reduction performance, this worst-case value represents the most unfavorable one-factor perturbation within the measured process-level tolerance range. The predicted *R_μ_*, Δ*R_μ_*, and relative increase under each perturbation condition are summarized in **Table 8**.

**Table 8:** Model-based one-factor local sensitivity analysis of the representative printed claw configuration under process-level angular and linear manufacturing deviations.

Perturbed combination	Parameter combination $\left(\theta ,\;L_{p},\;\gamma ,\;H_{t}\right)$	Predicted *R_μ_*	Δ*R_μ_*	Relative increase (%)
Nominal ***x***_0_	$\left(90.00^{\circ },\;16.00\text{ mm},\;75.00^{\circ },\;1.40\text{ mm}\right)$	1.0418	–	–
$x_{\theta ,\text{worst}}$	$\left(91.33^{\circ },\;16.00\text{ mm},\;75.00^{\circ },\;1.40\text{ mm}\right)$	1.0507	+0.0089	+0.85
$x_{L_{p},\text{worst}}$	$\left(90.00^{\circ },\;16.00\text{ mm},\;75.00^{\circ },\;1.40\text{ mm}\right)$	1.0418	0.0000	0.00
$x_{\gamma ,\text{worst}}$	$\left(90.00^{\circ },\;16.00\text{ mm},\;73.67^{\circ },\;1.40\text{ mm}\right)$	1.0876	+0.0458	+4.39
$x_{H_{t},\text{worst}}$	$\left(90.00^{\circ },\;16.00\text{ mm},\;75.00^{\circ },\;1.40\text{ mm}\right)$	1.0418	0.0000	0.00

***x***_0_ denotes the nominal representative printed claw configuration corresponding to the sixth run in the constrained D-optimal design. $x_{j,\text{worst}}$ denotes the one-factor worst-case combination obtained by perturbing only the *j*-th parameter. Δ*R_μ_* is the difference between the predicted *R_μ_* under $x_{j,\text{worst}}$ and the nominal value. A positive relative increase indicates reduced force-reduction performance.

**Figure 20:**
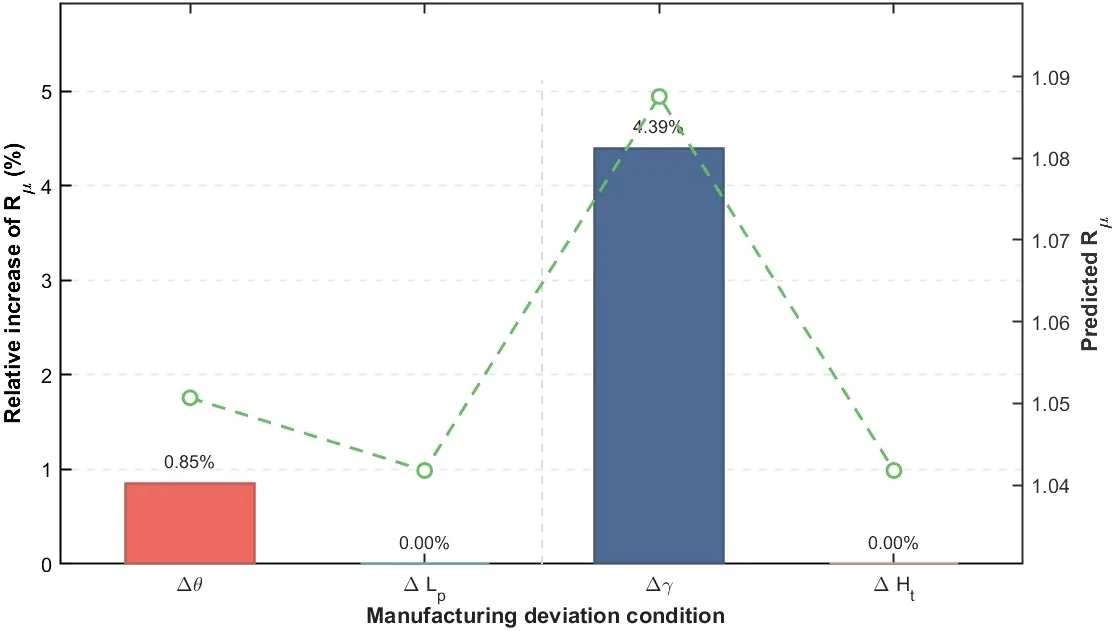
Predicted relative increase in *R_μ_* under measured local manufacturing deviations. A larger value indicates a greater reduction in force-reduction performance.

**Figure 20** visualizes the relative increase in *R_μ_* and the corresponding predicted *R_μ_* under each one-factor worst-case perturbation condition.

As shown in **Figure 20**, the predicted *R_μ_* changed only slightly within the measured manufacturing deviation range, indicating a certain local tolerance to manufacturing deviations. Among the four parameters, the apex angle *γ* was the most sensitive, increasing the predicted *R_μ_* by 4.39%, whereas the effect of *θ* was smaller, with an increase of 0.85%. The perturbations of *L_p_* and *H_t_* did not increase the predicted response within the truncated local tolerance intervals. Therefore, *γ* should be prioritized in subsequent printing, machining, and quality control.

#### 3.2.3 Performance comparison and validation experiments

Based on the independent validation test design described above, the actual critical shelling forces of the flat-press control group and the optimized claw group were first compared, as shown in **Table 9**. Then, another independent set of samples was used to further evaluate post-shelling kernel damage and biological viability, as summarized in **Table 10**.

**Table 9:** Validation test results and force-reduction performance compared with the flat-press control group.

Experimental group	Sample size	Measured mean force $\bar{F}$ [N]	Actual force reduction (%)
Flat-press Control Group	30	87.43	–
Optimized Group (*G_c_*,*_opt_*)	30	53.49	38.82

The actual force reduction percentage was calculated relative to the measured mean force of the flat-press control group, i.e., $\left({\bar{F}}_{\text{flat}}-{\bar{F}}_{\text{opt}}\right)/{\bar{F}}_{\text{flat}}\times 100\%$.

**Table 10:** Post-shelling kernel damage and biological viability evaluation.

Experimental group	Recovered kernels *N_t_*	*D*_0_	*D*_1_	*D*_2_	*D*_3_	Intact rate *P*_intact_ (%)	Visible damage *P*_visible_ (%)	Severe damage *P*_severe_ (%)	Germination rate *G_r_* (%)
Flat-press Control Group	50	42	3	2	3	84.00	16.00	10.00	92.00
Optimized Group (*G_c,opt_*)	50	47	3	0	0	94.00	6.00	0.00	98.00

*D*_0_–*D*_3_ denote the numbers of kernels classified as Grade 0–Grade 3 according to **Figure 16**. *P*_intact_, *P*_visible_, and *P*_severe_ were calculated according to the equations in Section 2. The germination rate was calculated based on the number of normally germinated kernels on the 5th day of incubation.

## 4 Discussion

### 4.1 Validation of the bending-dominated shelling mechanism

The mechanical premise of this study is that peanut shell rupture should be promoted by local bending-induced tensile stress rather than by excessive whole-pod compression. The 38.82% reduction in mean critical shelling force supports this assumption, indicating that the stress-concentration protrusion effectively enhanced local rupture while reducing the overall compression required before shell cracking.

The ANOVA results further support this mechanism. The significant interaction between wedge angle and protrusion height (*X*_1_*X*_4_), together with the significant quadratic effect of apex angle ( $X_{3}^{2}$), indicates that the shelling response was not governed by a single geometric factor. Instead, lateral constraint, contact dominance, and protrusion sharpness jointly affected crack initiation and propagation. The improved intact-kernel rate and germination rate also show that earlier local shell cracking reduced the high-risk compression interval in which the shell-kernel clearance decreases and the kernel becomes more likely to be compressed.

### 4.2 Methodological advantage of the MC-DORSO framework

A key difficulty in optimizing biological-material processing tools is that the measured response is affected not only by structural parameters but also by biological variability. In this study, pod size caused a significant baseline shift in absolute critical shelling force, whereas the interaction between mechanical configuration and size group was not significant. This result supports the use of the mean-normalized response *R_μ_* to reduce size-dependent baseline effects and to focus the regression model on geometry-related trends.

The constrained D-optimal component also improved the practical relevance of the experimental design. Conventional response surface designs may generate mathematically valid but mechanically infeasible parameter combinations. By embedding feeding-opening, orientation-envelope, seed-protection, and protrusion-mounting constraints into the candidate-space filtering process, the MC-DORSO framework restricted D-optimal sampling to a physically feasible design region. Therefore, the selected runs retained both statistical information content and engineering feasibility.

### 4.3 Engineering implications of the optimized configuration

The optimized configuration selected *θ* = 120°, *L_p_* = 16 mm, *γ* = 45°, and *H_t_* = 0.6 mm. This combination suggests that a wider wedge angle and wider flat surface helped reduce excessive lateral clamping, while a sharp but low protrusion mainly served to initiate local cracks rather than to penetrate deeply into the shell. This is consistent with the objective of reducing critical shelling force while limiting kernel damage risk.

The local tolerance sensitivity analysis showed that the predicted response changed only slightly within the measured manufacturing deviation range. Among the four parameters, the apex angle *γ* had the largest adverse effect on predicted *R_μ_*, whereas deviations in *L_p_* and *H_t_* did not increase the predicted response within the truncated local tolerance intervals. Therefore, future fabrication and quality control should prioritize the angular accuracy of the stress-concentration protrusion.

Therefore, the main contribution of this study is not a fixed claw geometry intended for all peanut varieties, but a re-parameterizable low-damage shelling design framework. For other varieties, pod dimensions, shell thickness, shell-kernel clearance, and kernel position should be remeasured, after which the same workflow can be used to update the feasible design space and optimize the claw parameters.

### 4.4 Limitations and future perspectives

This study was based on the “Luhua 11” peanut variety and single-pod quasi-static compression tests. Therefore, the optimized parameters should not be directly treated as universal dimensions for all varieties or as direct evidence of whole-machine performance. Future work should include more peanut varieties and extend the single-claw structure into a multi-array or continuous shelling mechanism. DEM–multibody dynamics (DEM–MBD) coupled simulations and dynamic shelling tests should also be conducted to evaluate shelling efficiency, kernel damage, and germination performance under practical high-speed shelling conditions.

## 5 Conclusions

To address the industrial challenges of poor size adaptability and excessive kernel damage in conventional peanut shelling equipment, this study proposes and validates a flexible shelling claw design leveraging lateral constraints and stress concentration mechanisms. By integrating mechanical analysis with statistical optimization, the main conclusions are as follows:

1. Establishment of a bending-dominated mechanical model: A HCBC model was developed based on pod morphology, indicating that shell rupture is a bending-dominated tensile mechanism triggered by radial compression. By reducing the local curvature radius at the contact point via a sharp protrusion, stress is effectively concentrated at the ventral suture. This significantly shortens the destructive axial compression stroke and accelerates Mode I (opening mode) fracture, thereby helping reduce kernel damage risk.
2. In the MC-DORSO framework, the mean-normalized index *R_μ_* was introduced to reduce baseline force differences among representative size groups. Combined with adaptive stepwise regression, this approach enabled the dominant geometric effects on *R_μ_* to be extracted from low-SNR biological-material experimental data and provided a transferable reference for similar structural optimization problems involving biological materials.
3. Geometry-constrained D-optimal experimental design: Compared with conventional D-optimal experimental design, the MC-DORSO framework embeds the derived geometric constraints into the D-optimal design and response surface optimization process to exclude infeasible or low-value candidate points. As a result, the D-optimal criterion is applied within the feasible domain to select experimental points with high information content. This reduces the likelihood of selecting mechanically invalid or low-information design points, allowing the response surface model to be built on feasible and informative experimental data and thereby improving the engineering reliability of the optimization results.
4. Simultaneous improvement in shelling performance and kernel preservation: Global optimization based on the relative shelling efficiency index *R_μ_* identified the optimal claw configuration. Physical validation showed that, within the representative dimensional variation range of the tested sample population, the optimized claw reduced the mean critical shelling force by 38.82% compared with the flat-press control group. Combined with the geometric constraint boundary *D*_fix_, this mechanical optimization helps reduce the probability of excessive shell flattening and kernel compression before effective shell rupture. Additional post-shelling damage and germination tests further showed that, under the quasi-static loading conditions used in this study, the optimized claw improved kernel preservation, as reflected by lower mechanical damage and better germination performance. Therefore, this structure provides a feasible mechanical design strategy for low-damage precision shelling of high-value seed peanuts.

Overall, the proposed method reduced the load required for single-pod quasi-static shell rupture within the representative dimensional variation range and provides a scalable basic shelling unit and optimization method for future low-damage seed peanut shelling mechanisms.

## Data Availability

The raw data supporting the conclusions of this article will be made available by the authors, without undue reservation.
