# Supplementary material for: Mechanism-constrained D-optimal design of a flexible claw for peanut shelling under biological variability
Source: Front Plant Sci. 2026 Aug 17;17:1831721. doi: 10.3389/fpls.2026.1831721 (PMC13527014; doi:10.3389/fpls.2026.1831721)
Supplement: Supplementary file 1 [file Table1.pdf]

Supplementary Tables S1–S4

Supplementary Material

Supplementary Material for:
Mechanism-constrained D-optimal design of a flexible claw for peanut shelling under biological variability

Manuscript DOI: 10.3389/fpls.2026.1831721

Contents

Appendix A. Nomenclature **3**

Appendix B. MC-DORSO implementation workflow and physical interpretation of constraints **7**

Appendix A. Nomenclature

Supplementary Table S1: Abbreviations used in this study.

Abbreviation	Full term
MC-DORSO	Mechanism-constrained D-optimal response surface optimization
HCBC	Hertzian-curved beam coupling
RSM	Response surface methodology
SNR	Signal-to-noise ratio
FEM	Finite element method
DEM	Discrete element method
ANOVA	Analysis of variance
PLA	Polylactic acid
CAD	Computer-aided design
RMSE	Root mean square error

Supplementary Table S2: Nomenclature used in this study.

Symbol	Definition	Unit
a	Minor-axis dimension of the peanut pod cross-section	mm
b	Major-axis dimension of the peanut pod cross-section	mm
A_1/A_2, B_1/B_2	Endpoints of the minor and major axes, respectively	–
I	Stress-concentration point marked in the mechanism schematic	–
s	Ventral suture loading plane	–
t	Average shell thickness	mm
g, $g/2$	Shell-kernel clearance and half-clearance	mm
$a_{\max}$	Maximum minor-axis dimension observed in the pre-test sample population and used for the conservative feeding-opening constraint	mm
$b_{\min}$, $b_{\max}$	Minimum and maximum major-axis dimensions of the screened peanut pods	mm
$g_{\min}$	Minimum shell-kernel clearance obtained from the pre-test	mm
$t_{\min}$	Minimum shell thickness obtained from the pre-test	mm
$\Delta A/2$, $\Delta B/2$	Lateral expansion and vertical shortening on each side after compression	mm
δ_a	Outward deformation along the minor-axis direction of the peanut pod	mm
δ_b	Inward deformation along the major-axis direction of the peanut pod	mm
l	Pod apex or crack-initiation apex	–
F_s	Total externally applied shelling or gripping force	N
F	Applied normal load in the Hertz contact model	N
F_N	Normal force acting on the inclined surface	N
F_x	Lateral compression force generated by the inclined surfaces	N

Symbol	Definition	Unit
q_s	Equivalent radial load per unit shell width, $q_s = F_s/w_s$	$\mathrm{N\,mm^{-1}}$
w_s	Effective shell width involved in local deformation	mm
σ_N	Direct compressive stress component	MPa or $\mathrm{N\,mm^{-2}}$
m_s	Bending moment per unit shell width	N
σ_M	Bending-induced tensile stress component	MPa or $\mathrm{N\,mm^{-2}}$
ζ	Dimensionless local bending geometry coefficient	–
σ_{in}	Resultant circumferential stress on the inner shell wall	MPa or $\mathrm{N\,mm^{-2}}$
σ_{bond}	Interlaminar bond strength of the fibrous layer	MPa or $\mathrm{N\,mm^{-2}}$
$\sigma_{\max}$	Maximum local stress near the stress-concentration tip	MPa or $\mathrm{N\,mm^{-2}}$
σ_{yield}	Yield stress used for rupture initiation description	MPa or $\mathrm{N\,mm^{-2}}$
R_{eq}	Equivalent local curvature radius of the peanut shell	mm
ρ_{major}, ρ_{minor}	Auxiliary curvature radii used in the elliptical approximation	mm
R_{ind}	Curvature radius of the loading object or contact indenter	mm
R^*	Relative radius of curvature in the Hertz contact model	mm
$p_{\max}$	Maximum Hertzian contact pressure	MPa or $\mathrm{N\,mm^{-2}}$
E^*	Equivalent elastic modulus of the contact pair	MPa
L	Contact length in the Hertz contact model	mm
θ	Wedge angle of the V-shaped guiding surfaces	degree
L_p	Flat surface width of the claw	mm
D	V-groove depth or enveloping depth	mm
D_{design}	Designed V-groove depth calculated from the safety and compensation terms	mm
D_{fix}	Fixed V-groove depth used in the experimental claw design	mm
D_{limit}	Conservative reference boundary for seed-protection depth	mm
L_d	Top opening distance of the V-shaped guiding region	mm
ΔL_d	Single-side contribution to the top opening distance	mm
α	Instantaneous posture angle between the peanut major axis and the horizontal x-axis in the claw-opening plane	degree
$W_{\mathrm{env}}(\theta, \alpha)$	Lateral envelope width required by the tilted pod during orientation adjustment	mm
$L_{p,C2}^{\mathrm{env}}(\theta)$	Orientation-envelope lower bound of L_p after eliminating the posture variable α	mm
x, $2\lvert x\rvert$	Half and full lateral envelope width required by a tilted peanut pod	mm
M	Shelling stroke	mm

Symbol	Definition	Unit
η	Reliability derating factor	–
γ	Apex angle of the stress-concentration protrusion	degree
H_t	Height of the stress-concentration protrusion	mm
W_t	Theoretical basal width of the protrusion	mm
K_{shell}	Equivalent constraint stiffness of the shell	N mm^{-1}
K_t	Stress concentration factor	–
G_c	Control-factor set, $G_c = \{\theta, L_p, \gamma, H_t\}$	mixed
$G_{\text{c,opt}}$	Optimal control-factor configuration	mixed
$G(a, b)$	Pod-size noise factor defined using a and b	–
L_1, L_2, L_3	Representative pod-size levels for the noise factor $G(a, b)$: small, medium, and large groups	–
μ, σ	Mean and standard deviation of measured variables	variable-dependent
X_1, X_2, X_3, X_4	Design variables corresponding to θ, L_p, γ, and H_t, respectively	mixed
Y	Response used in the design-stage regression basis	variable-dependent
β	Regression coefficient in the response surface model	variable-dependent
ϵ	Regression error term	variable-dependent
Ω	Feasible design region for constrained D-optimal sampling	–
C_1, C_2^{env}, C_3, C_4	Feeding-opening, orientation-envelope, seed-protection, and protrusion-mounting constraints	–
$\mathbf{X}$	Design matrix used in the D-optimality criterion $\lvert\mathbf{X}^T\mathbf{X}\rvert$	–
N_{run}	Number of D-optimal experimental runs	count
N_{state}	Number of aggregated physical states used for response surface modeling	count
$\mathcal{C}_0$	Full discrete candidate set before constraint filtering	–
$\mathcal{C}$	Feasible candidate set after constraint filtering	–
$\mathbf{X}_{\text{D}}$	Selected D-optimal design matrix	–
$R_{\mu,rkj}$, R_μ	Mean-normalized relative shelling efficiency index	–
$F_{\text{crit},rkj}$	Critical shelling force of the j-th replicate in the k-th pod-size group under the r-th claw configuration	N
$\bar{F}_k$	Mean critical shelling force of the k-th pod-size group	N
Δ_{ang}, Δ_{lin}	Angular and linear manufacturing deviations	degree, mm
A^{meas}, A^{nom}	Measured and nominal angular dimensions	degree
L^{meas}, L^{nom}	Measured and nominal linear dimensions	mm
Δ_j	Manufacturing deviation assigned to the j-th design parameter	degree or mm
$X_{j,0}$	Nominal value of the j-th design parameter	degree or mm
$X_{j,\min}$, $X_{j,\max}$	Lower and upper bounds of the j-th design parameter	degree or mm
$X_{j,\text{low}}$, $X_{j,\text{up}}$	Truncated lower and upper perturbation bounds of the j-th parameter	degree or mm

 DOI: 10.3389/fpls.2026.1831721

Symbol	Definition	Unit
$\mathcal{C}_j$	Candidate perturbation set for the j-th parameter	–
$X_{j,\mathrm{worst}}$	One-factor worst-case perturbation value of the j-th parameter	degree or mm
$\mathbf{x}_0$	Nominal representative printed claw configuration	mixed
$\mathbf{x}_{j,\mathrm{worst}}$	One-factor worst-case perturbed configuration	mixed
D_0–D_3	Numbers of kernels classified as damage Grades 0–3	count
N_t	Total number of recovered target kernels that could be evaluated	count
P_{intact}	Intact-kernel rate	%
P_{visible}	Visible mechanical damage rate	%
P_{severe}	Severe mechanical damage rate	%
G_r	Germination rate	%
N_{germ}	Number of normally germinated kernels	count

 DOI: 10.3389/fpls.2026.1831721

Appendix B. MC-DORSO implementation workflow and physical interpretation of constraints

Supplementary Table S3: Implementation workflow of the mechanism-constrained D-optimal response surface optimization (MC-DORSO) framework

Phase	Step	Operation	Key criterion or input	Output
I	1	Define morphology distribution and mechanical design variables	$G(a,b)$, g, t, $G_c = \{\theta, L_p, \gamma, H_t\}$	Initial parameter space
I	2	Establish geometric and mechanical feasibility boundaries	D_{fix}, factor ranges, and constraints C_1, C_2^{env}, C_3, and C_4	Feasible-space rules
II	3	Generate discrete candidate points within the full factor ranges	Grid discretization of θ, L_p, γ, and H_t	Full candidate set $\mathcal{C}_0$
II	4	Apply Boolean filtering to each candidate point	Feeding, orientation-envelope, seed-protection, and mounting constraints	Feasible candidate set $\mathcal{C}$
II	5	Construct the candidate design matrix using the design-stage regression basis	Main effects, selected interactions, and quadratic terms	Candidate model matrix
III	6	Initialize a 16-run design from the feasible candidate set	Fixed random seed `rng(10, 'twister')`	Initial design matrix
III	7	Perform coordinate exchange and evaluate each trial replacement	Maximize $\lvert \mathbf{X}_{\mathrm{D}}^T \mathbf{X}_{\mathrm{D}} \rvert$	Updated design matrix
III	8	Check convergence of the D-optimal search	No further increase in the information determinant	Constrained 16-run D-optimal design
IV	9	Conduct shelling tests and construct the mean-normalized response	$R_{\mu,rkj} = F_{\text{crit},rkj}/\bar{F}_k$	Response dataset
IV	10	Fit the reduced response surface and perform constrained parameter optimization	Adaptive stepwise regression and mixed discrete–continuous optimization	Optimal claw configuration

Supplementary Table S4: Physical interpretation of the design constraints used in the constrained D-optimal design

Constraint	Main variables	Practical role	Consequence if violated
C_1 Feeding constraint	θ, L_p	Defines the effective entrance width of the V-shaped guiding region.	Large pods may jam or fail to enter the gripping zone.
C_2^{env} Orientation-envelope check	θ, L_p (projected over α)	Evaluates whether the selected θ–L_p window provides sufficient lateral envelope width after the uncontrolled posture angle α has been eliminated through projection.	The pod may not be sufficiently accommodated by the guiding opening, and the ventral suture may fail to align with the central protrusion.
C_3 Seed-protection constraint	H_t	Limits protrusion height to reduce intrusion into the kernel-occupied region.	The protrusion may increase the risk of kernel compression or injury.
C_4 Mounting constraint	L_p, H_t, γ	Ensures that the protrusion base fits within the flat surface.	The protrusion may become geometrically infeasible or interfere with the V-shaped surfaces.
